# Factors affecting the healthcare utilization from Shasthyo Suroksha Karmasuchi scheme among the below-poverty-line population in one subdistrict in Bangladesh: a cross sectional study

**DOI:** 10.1186/s12913-022-08254-1

**Published:** 2022-07-08

**Authors:** Md. Zahid Hasan, Mohammad Wahid Ahmed, Gazi Golam Mehdi, Jahangir A. M. Khan, Ziaul Islam, Mahbub Elahi Chowdhury, Sayem Ahmed

**Affiliations:** 1 Health Systems and Population Studies Division, Health Economics and Financing, icddr,b, 68 Shahid Tajuddin Ahmed Sharani, Mohakhali, Dhaka 1212 Bangladesh; 2 Leeds Institute of Health Sciences, University of Leeds, 6 Clarendon Way, Woodhouse, LS2 9NL Leeds, UK; 3 Health Economics and Policy Unit, School of Public Health and Community Medicine, University of Gothenburg, Medicinaregatan 18A, 405 30 Gothenburg, Sweden; 4 Health Economics and Health Technology Assessment (HEHTA), Institute of Health & Wellbeing, University of Glasgow, 1 Lilybank Gardens, Glasgow, G12 8RZ UK

**Keywords:** Health protection scheme, Utilization of healthcare, Below poverty line population, Bangladesh

## Abstract

**Background:**

Financing healthcare through out-of-pocket (OOP) payment is a major barrier in accessing healthcare for the poor people. The Health Economics Unit (HEU) of the Ministry of Health and Family Welfare of the government of Bangladesh has developed *Shasthyo Suroksha Karmasuchi* (SSK), a health protection scheme, with the aim of reducing OOP expenditure and improving access of the below-poverty-line (BPL) population to healthcare. The scheme started piloting in 2016 at Kalihati sub-district of Tangail District. Our objective was to assess healthcare utilization by the enrolled BPL population and to identify the factors those influencing their utilization of the scheme.

**Method:**

A cross-sectional household survey was conducted from July to September 2018 in the piloting sub-district. A total of 806 households were surveyed using a semi-structured questionnaire. Information on illness and sources of healthcare service were captured for the last 90 days before the survey. Multiple logistic regression models were applied to determine the factors related to utilization of healthcare from the SSK scheme and other medically trained providers (MTPs) by the SSK members for both inpatient and outpatient care.

**Result:**

A total of 781 (24.6%) people reported of suffering from illness of which 639 (81.8%) sought healthcare from any sources. About 8.0% (51 out of 639) of them sought healthcare from SSK scheme and 28.2% from other MTPs within 90 days preceding the survey. Households with knowledge about SSK scheme were more likely to utilize healthcare from the scheme and less likely to utilize healthcare from other MTPs. Non-BPL status and suffering from an accident/injury were significantly positively associated with utilization of healthcare from SSK scheme.

**Conclusion:**

Among the BPL population, healthcare utilization from the SSK scheme was very low compared to that of other MTPs. Effective strategies should be in place for improving knowledge of BPL population on SSK scheme and the benefits package of the scheme should be updated as per the need of the target population. Such initiative can be instrumental in increasing utilization of the scheme and ultimately will reduce the barriers of OOP payment among BPL population for accessing healthcare.

**Supplementary Information:**

The online version contains supplementary material available at 10.1186/s12913-022-08254-1.

## Introduction

Universal health coverage (UHC) is now a familiar acronym for the global health agenda that combines a triple policy. It is defined as ensuring universal access to quality healthcare services as per need without the risk of incurring financial hardship [1]. More recently, the adaption of the sustainable development goals (SDGs) by the United Nation assembly explicitly included the UHC goal under SDG 3.8 stating "Achieve Universal Health Coverage, including financial risk protection, access to quality essential healthcare services, and access to safe, effective, quality, and affordable essential medicines and vaccines for all" [2]. Access to healthcare is considered as an important determinant in assessing equity in healthcare delivery [3]. Ideally, the use of healthcare services reflects the need for care; however, that is not always possible for several reasons [4]. In low- and middle-income countries (LMICs), healthcare utilization is influenced by demand and supply-side constraints. In some instances, utilization is determined solely by the ability to pay or out-of-pocket (OOP) payments rather than the need for care [5]. This situation can gradually impose heavy financial burdens on individuals as well as on households and consequently can lead to catastrophic healthcare expenditure (CHE) and economic impoverishment [6, 7]. Like other LMICs, the healthcare financing strategies in Bangladesh employs a combination of general revenue taxation, OOP payments, development partner’s contribution, corporate insurance, and others. OOP spending is the main source of healthcare financing in Bangladesh and the share of OOP payments has increased from 55.9% in 1997 to 67% in 2015 [8]. Reliance on OOP payments for financing healthcare and significantly increasing treatment cost related to non-communicable disease and epidemiological transition (responsible for 60% of the mortality) have led to increase the OOP healthcare spending in Bangladesh. Such a high percentage of OOP expenditure for healthcare is associated with low financial protection of the households. A study using data from 2010, revealed that 14.7% of the households faced CHE from OOP healthcare payments and 3.5% of the total population falls into poverty annually [9]. Another study conducted among 11 Asian countries in 2006 showed that about 5 million people become impoverished annually in Bangladesh from OOP healthcare expenditure [10].

Public spending on health is determined by the capacity of the government to raise revenues and allocate these to the health sector [11]. Bangladesh spends only 3% of its gross domestic product (GDP) in health sector while government expenditure compared to GDP is only 0.69%. Such spending has placed Bangladesh on the list of countries with the lowest health spending in the South-East Asian Region [12].

The latest household income and expenditure survey 2015 of Bangladesh reported that among those who access healthcare, 55.62% utilize services from medically non-trained provider’ (non-MTPs) which includes non-qualified private practitioner, traditional healer, and drug sellers [13]. People prefer to seek health care from non-MTPs in the informal sector, especially the poor and the disadvantaged [14] rather visiting medically trained providers (MTPs) such as medical college hospitals, Upazila Health Complex (UzHC), general practitioners, non-government organization (NGO) hospitals, qualified private practitioners, Union Sub-Centres (USC), and Maternal and Child Welfare Centres (MCWC).

The 2011 National Health Policy of the Government of Bangladesh (GoB) acknowledges that health is a human right, and to achieve UHC, it is necessary to ensure health services for the poor at an affordable cost [15]. Effective measures must be taken to reduce households’ OOP healthcare expenditure and increase utilization of quality healthcare to achieve UHC by 2030. Responding to the global call for UHC, the GoB adopted the first-ever Health Care Financing Strategy in 2012 aiming to achieve UHC by 2032 [16]. Under this strategy, the Shasthyo Surokhsha Karmasuchi (SSK), a GoB health protection scheme, is being piloted by the Health Economics Unit (HEU) of the Ministry of Health and Family Welfare [17]. Although the SSK scheme has a comprehensive plan to bring all the population of the country under health insurance, initially it aimed to pilot only among the below-poverty-line (BPL) population.

### Description of Shasthyo Surokhsha Karmasuchi (SSK)

SSK scheme is a model of health insurance that started piloting at Kalihati Upazila of Tangail District in 2016 among the BPL households. Later in 2017, the scheme expanded to two other subdistricts (upazilas) – Modhupur and Ghatail of the same district. Currently, total 96,432 BPL households from the three subdistricts are enrolled in the SSK scheme. The BPL households of the scheme were selected based on following selection criteria i.e., household head is a regular day labour, have no land except dwelling place, and no permanent income source. If a household meets any two conditions of the mentioned criterion, the household was listed as BPL and entitled to get the scheme benefits. The scheme provides primary inpatient and outpatient care to the enrolled BPL households through the UzHC with a structured referral to the secondary level district hospital at Tangail for complicated cases.

In the UzHC, non-SSK patients also can seek healthcare without getting benefits of SSK scheme i.e., free inpatient and outpatient care. Generally, people need to pay fee for getting consultation and admission at the UzHC. In addition, all prescribed drugs and diagnosis for regular patients are not available. However, under the SSK scheme, enrolled households get inpatient care without incurring any expenditure for registration, admission, drugs, and diagnosis. The SSK enrollees get outpatient consultation without fees and drugs from regular hospital supplies. The scheme contracted private providers for suppling drugs and diagnostic services to the SSK patients. It is noted that only SSK inpatient gets all types of medicines and diagnosis services from the contracted private providers. The SSK outpatients get medicines and diagnosis as general outpatients do.

Each member household is provided an electronic health card. The validity of the card is one year, and the membership is planned to update every year. This card ensures that the enrolled households will get coverage of BDT 50,000 per year for inpatient care (consultation, drugs, and diagnosis) for 78 different disease groups and outpatient consultation only against a government-financed premium of BDT 1,000 per household per year. At the initial stage of scheme implementation, the HEU developed an information, education, and communication strategy for promoting awareness and healthcare utilization by the enrolled population. The strategy includes continuation of loudspeaker announcement, production and telecasting messages through local cable network, poster and leaflet distribution, and union level meeting with identified population. The scheme is financed through HEU generated pool fund using the allocated premium from the government for the BPL households. The UzHC and district hospital are reimbursed from this fund for providing free healthcare to the SSK patients based on the submitted verifiable patient records (claim). Reimbursement follows a case and diagnosis-based payment system using a simplified diagnosis related group (DRG) system. The following table provides an overview of facilities at the government subdistrict level UzHC with SSK scheme and without SSK scheme (Table 1). A detailed description of the scheme is published elsewhere [18].

**Table 1:** Differences of benefits between patients with SSK and without SSK at SSK UzHC

Patients with SSK	Patients without SSK
- Free inpatient and outpatient consultation - Free drugs and diagnostic services for 78 disease groups for inpatient care from contracted private sources - Structured referral from UzHC to district hospital with transportation support - Free healthcare supports up to BDT 50,000 per enrolled household per year - Premium BDT 1,000 per household per year paid by the government	- Need to pay for inpatient and outpatient consultation - Not all drugs and diagnostics are available at regular supply of hospital - Need to pay for all types of diagnostics services - No structured referral with transportation support - No facility of insurance coverage and premium

Health insurance schemes are viewed as an alternative to improving access to healthcare and reducing the direct financial burden to the patients while using health services [19, 20]. A subsidized health insurance scheme in Combodia reduced OOP payment and increased healthcare utilization among its beneficiaries [21, 22]. The Askeskin program in Indonesia improved access of its members to healthcare facilities and increased outpatient utilization [23, 24]. A publicly financed health protection scheme in India increased hospital services utilization by the beneficiaries and it sustained over time and across the regions [25]. Studies shows that several factors including age, sex, marital status, types of illness, household size, occupation, income or socioeconomic status, and understanding of insurance determine the utilization of healthcare from such health protection scheme [26–28].

However, empirical evidence on healthcare utilization among the members of the SSK scheme is not available. The GoB is planning to expand this scheme in some other upazilas of the same district as well as gradually to all over the country. In this context, evidence on the use of SSK scheme and healthcare seeking behaviour of the enrolled households’ members will help policymakers to understand the influencing factors and plan the implementation accordingly. Therefore, our objective was to assess the utilization of healthcare among the enrolled BPL population and identify the factors that influenced their use of healthcare from the SSK scheme and other MTPs.

## Methods

### Theoretical framework of healthcare utilization

**Fig. 1:**
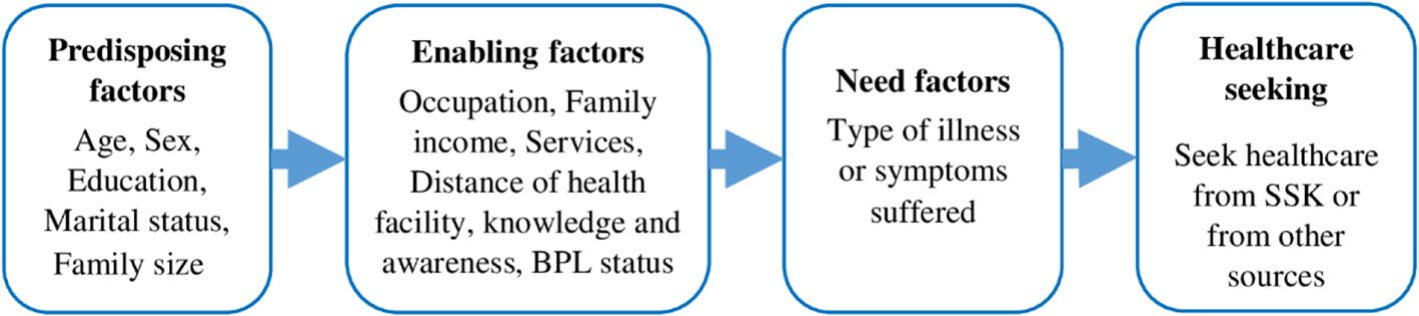
Andersen’s Behavioral Model of Health Service Utilization (Modified)

It is evident in the literature that the healthcare utilization model developed by Andersen is a well-validated theoretical framework aimed at understanding the determinants of health service utilization. The framework provides a systematic analysis on the societal and individual determinants of healthcare service utilization. According to the model, three different set of factors influence individuals’ decisions to utilize healthcare services e.g., predisposing, enabling, and need factors [29]. In this study, predisposing factors include the sociocultural characteristics of the individual such as age, sex, marital status, education, and household size, which may increase the need for health services. Enabling factors facilitate or impede the use of health service which includes, occupation, distance from the health facility, knowledge and awareness about the scheme, current BPL status, and family income. The need factors represent both perceived need and actual need, such as the view and experience of the individual about the illness they suffered from. Figure 1 shows Andersen’s behavioral model of health service utilization to examine the influence of different factors on the use of healthcare from SSK scheme and from other MTPs by the BPL population. In addition to the SSK scheme, we classified sources of healthcare into two other categories; one is other MTPs, which includes all sources of MTPs excluding the SSK UzHC and non-MTPs. Our outcome variable was the utilization of care from SSK scheme (SSK UzHC) or from other MTPs in the last 90-day.

### Study design, settings, and population

This was an exploratory study carried out in Kalihati Upazila (subdistrict) of Tangail District where the SSK was being piloted. The total number of households in this Upazila is 89,351 of which 35,740 (40%) households were identified as BPL and enrolled in the SSK scheme.

### Data collection

A cross-sectional household survey was conducted using a pretested semi-structured questionnaire (supporting information 1). Information on household's socio-demographic characteristics, history of illness and treatment, care seeking from different types of healthcare providers, and knowledge and awareness about the SSK scheme was collected through the survey. In each household, the head of the household was selected as respondent. In an absence of household head while visiting the household, an individual aged above 18 and had knowledge about the other members of the household was selected as respondent. A six-member team of trained and experienced (5 data collectors and a supervisor) enumerator was employed in data collection. Informed written consent from the respondents was obtained before enrolling them in the survey. The survey was conducted from July to September 2018 and a 90-day recall period was considered for data collection.

### Sampling

The sample size was determined based on the use of a similar health protection scheme among the BPL population called *Rashtriya Swasthya Bima Yojana* (RSBY) in India [27]. Using 19% utilization of the scheme, considering 95% confidence interval, and 3% error level, a minimum of 657 households were required to interview. Assuming 5% nonresponse rate and 1.2 design effect for clustering, a total of 828 BPL households were finally selected for the survey.

The BPL household list of the SSK scheme was collected from SSK programme and was used as a sampling frame for the survey. The households in the Kalihati upazila were selected following two-stage cluster sampling method. There are 15 Unions (collection of villages), in Kalihati Upazila. In the first phase of sampling, the Unions were classified into three subgroups i.e., nearer (up to 6 km), medium (7 to 15 km), and far (More than 15 km) based on the distance of the Unions to the UzHC; the first point of care for SSK members. In each of the 15 Unions, the number of enrolled BPL households were unequal. Therefore, the number of BPL households to be interviewed from a Union was determined according to the proportion of total BPL households from that Union. In the second phase, a simple random sampling technique was used to select the required number of BPL households from the BPL lists for each Union. However, while visiting the sampled SSK households, we found that some of the households did not receive SSK cards yet and were not eligible to receive healthcare under the scheme. In such cases, we interviewed the adjacent households who had SSK cards.

### Analysis

The healthcare utilization behavior was assessed considering several dimensions for example types of illness suffered, type of providers utilized for both inpatient and outpatient care, inpatient care utilization status of the households, and sources of care. Inpatient was defined as one who required admission and stayed in the hospital at least an overnight and outpatient was defined as patients who did not require admission to the hospital or required admission but did not stay an overnight.

The descriptive characteristics of the surveyed households and members of the households were presented in frequency (n) and percentages (%) with a 95% confidence interval (CI). Households’ monthly income was estimated by combining the income of all individuals of an households and income from different sources e.g., income from land, sharecropping, fixed assets, and allowance or stipend. Some of the income items were reported for a year. In those cases, we converted those yearly income into monthly income by dividing with 12. The households were classified into five quintiles based on their total monthly income using STATA 16. It might happen that some of the enrolled households either have graduated from the BPL status to non-BPL or were not properly enrolled into the scheme. Thus, applying the same BPL selection criterion those were used by the SSK programme, we checked the current BPL status of the surveyed households. Household’s knowledge on the SSK scheme and sources of information related to SSK were assessed using the information from the respondents. The respondents were asked whether they knew about the SSK scheme and its service delivery system and what were their sources of knowledge.

We categorized the illnesses into communicable (e.g., Tuberculosis, Diarrhoea, Jaundice), non-communicable (e.g., Asthma, Hypertension, Diabetes, Arthritis), Accident/injury (e.g., Minor injury, bone fracture, and other accidental injury), Female reproductive and child delivery (e.g., Obstetric related health problem and delivery), symptoms (e.g., cough, fever, weakness, pian and discomfort), and Others (e.g., polips, scars). Two multiple logistic regression models were used separately to identify the predictors of healthcare utilization from the SSK scheme and from MTPs other than SSK UzHC. In the first model, the binary dependent variable was ‘Status of healthcare utilization from the SSK scheme (1 = utilized SSK scheme 0 = did not utilize SSK scheme)’ and in the second model the dependent variable was ‘MTP utilization other than SSK (1 = utilized care from MTPs other than SSK, 0 = did not utilize MTPs)’. Age, sex, marital status, occupation, education of members, types of self-reported illness, household size, at least one household member heard about the SSK scheme, current BPL status of the households, and distance of Union from the SSK facility were included as independent predictor variables. A *p* value of < 0.05 was considered as statistically significant.

### Ethics statement

We obtained informed written consent from all respondents who were enrolled into this study and ensured confidentiality and anonymity of their information. This study was approved by the Research Review Committee and the Ethics Review Committee of the International Centre for Diarrhoeal Disease Research Bangladesh (icddr,b) (Protocol# PR-17047).

## Results

### Socio-demographic characteristics of households and members

Table 2 shows the socio-demographic characteristics of households and members. Among the selected 828 households, 806 households were finally interviewed (97% response rate). These households had total 3,178 members of which majority were aged between less than 18 years to 34 years (59.3%) followed by 45 to 60 years (19.4%), 35 to 44 years (15.2%), and more than 60 years (6.0%) (Table 2). The proportion of male to female was almost equal (50.2% vs 49.8%). More than half of these members were married. Most of the members (37%) had no institutional education, while 27.5% had primary, and 25.5% had secondary or above level education. About 27% of the household members were housewives and around 15% were agricultural workers or day labors.

**Table 2:** Background characteristics of the members and households

Characteristics of members (*n* = 3,178)	n	%	95% CI
**Age group**			
Fewer than 18 years	1,131	35.6	(33.9–37.3)
18 to 34 years	754	23.7	(22.3–25.2)
35 to 44 years	483	15.2	(14.0–16.5)
45 to 60 years	618	19.4	(18.1–20.9)
> 60 years	192	6.0	(5.3–6.9)
**Sex**			
Male	1,595	50.2	(48.4–51.9)
Female	1,583	49.8	(48.1–51.6)
**Marital status**			
Married	1,754	55.2	(53.5–56.9)
Unmarried	972	30.6	(29.0–32.2)
Widowed/separated/destitute	128	4.0	(3.4–4.8)
Not applicable (age < = 5 years)	324	10.2	(9.2–11.3)
**Education of household members**			
No education	1,173	36.9	(35.2–38.6)
Up to Primary level	870	27.4	(25.9–29.0)
Secondary level and above	811	25.5	(24.0–27.1)
Not applicable (age < = 5 years)	324	10.2	(9.2–11.3)
**Occupation of household members**			
Housewife	863	27.2	(25.6–28.7)
Student	824	25.9	(24.4–27.5)
Agricultural/labor	467	14.7	(13.5–16.0)
Service worker	188	5.9	(5.1–6.8)
Small business	180	5.7	(4.9–6.5)
Unemployed	217	6.8	(6.0–7.6)
Transport worker	115	3.6	(3.0–4.3)
Not applicable (age < = 5 years)	324	10.2	(9.2–11.3)
**Reported any illness or symptoms**			
Yes	781	24.6	(23.1–26.1)
No	2397	75.4	(73.9–76.9)
**Characteristics of households (*n* = 806)**			
**Household size**			
Less than 4 persons	295	36.6	(33.3–40.0)
4—5 persons	425	52.7	(49.3–56.2)
6 persons or more	86	10.7	(8.7–13.0)
**Distance of health facility from union**			
Nearer (less than 6 km)	189	23.5	(20.6–26.5)
Medium (7 to 15 km)	328	40.7	(37.3–44.1)
Far (More than 15 km)	289	35.9	(32.6–39.2)
**Monthly income quintile (in BDT) **^**a**^			
< = 8000	181	22.5	(19.7–25.5)
> 8000 to < = 10,250	142	17.6	(15.1–20.4)
> 10,250 to < = 14,000	170	21.1	(18.4–24.1)
> 14,000 to < = 18,350	155	19.2	(16.7–22.1)
> 18,350	158	19.6	(17.0–22.5)

^**a**^ 1 USD = 83.7 Bangladeshi Taka; June 2018, Bangladesh Bank

From a household perspective, most of the households (52.7%) had 4–5 members, followed by less than 4 members (36.6%), and more than six members (10.7%). Around 36% of households were from unions of more than 15 km away from the SSK UzHC, while around 24% were nearer (less than 6 km) to the SSK UzHC, and majority (41%) were from the medium distance unions (7 to 15 km). More than one-third of households had total income less than BDT 10,250 (USD 122) per month.

### Households’ BPL status and knowledge of SSK scheme

Applying BPL household selection criterion, we found about 66% of the SSK households remained as BPL at the time of interview (Table 3). Regarding the knowledge of SSK scheme, about 68% of the surveyed BPL households knew about the SSK scheme. A higher proportion of respondents knew about the service provision of SSK scheme (63%), however, their knowledge about benefits of the SSK scheme was found to be poor. The majority (56%) of the beneficiaries reported that they learnt about SSK scheme from SSK representatives, followed by 25% from familiar people in locality, and 17% from relatives.

**Table 3:** Households' current BPL status, knowledge, and sources of information about the SSK scheme (*n* = 806)

	***n* = 806**	**%**	**95% CI**
**Current BPL status**			
BPL	529	65.6	(62.3–68.8)
non-BPL	277	34.4	(31.2–37.7)
**Heard about SSK scheme**			
Yes	546	67.7	(64.4–70.9)
No	260	32.3	(29.1–35.6)
**Knowledge on SSK service provision**			
Through card	508	63.0	(59.6–66.3)
Don't know	298	37.0	(33.7–40.4)
**Knowledge on treatment expenses at the SSK**^**a**^			
Free outpatient ticket	361	44.8	(41.4–48.2)
Free medicine for IPC	358	44.4	(41–47.9)
Free diagnostic test for IPC	154	19.1	(16.5–22)
Free referral facility to district hospital	76	9.4	(7.6–11.7)
Free treatment at referral DH	71	8.8	(7–11)
**Source of information**^**a**^			
Representative from SSK	527	55.9	(92.6–96.4)
Familiar people in locality	232	24.6	(37.7–45.9)
Relative	160	17.0	(25.2–32.7)
Local leader	16	1.7	(1.8–4.7)
Other sources (Audio broadcasting, poster, TV)	8	0.9	(0.7–2.9)

^a^ Multiple responses

### Utilization of healthcare

Table 4 presents the utilization of healthcare of the BPL population. Almost 25% of the surveyed people (*n* = 3,178) reported of having symptoms or illness within 90 days before the survey. Among the sick 25% (781) individuals, 81.8% (639) sought healthcare for their illnesses or symptoms from any type of healthcare provider. Only 8% of these individuals sought healthcare from the SSK scheme and 28.2% from MTPs other than SSK, and the rest (63.8%) of them went to non-MTPs. Majority of the individuals utilized healthcare from pharmacy (45.9%), followed by public providers (20.7%), and private providers (18.9%). Among the patients who sought healthcare, 3.6% (23) of them had inpatient care and 10 inpatients utilized healthcare from SSK provider. Of the total care seeker patients, 6.4% utilized outpatient care from the SSK provider and 26.1% from other MTPs.

**Table 4:** Pattern of healthcare utilization of the study population who reported any illness in the last 90-day (*n* = 781)

Characteristics	n	%	95% CI
**Types of self-reported illness/symptoms**			
Non-communicable	178	22.8	(20.0–25.9)
Communicable	103	13.2	(11.0–15.8)
Accident/Injury	29	3.7	(2.6–5.3)
Female reproductive	22	2.8	(1.9–4.2)
Symptoms	434	55.6	(52.1–59.0)
Others	15	1.9	(1.2–3.2)
**Sought healthcare among those who suffered**			
Yes	639	81.8	(78.9–84.4)
No	142	18.2	(15.6–21.1)
**Source of healthcare**			
SSK provider	51	8.0	(6.1–10.4)
Medically trained providers (non-SSK)	180	28.2	(24.8–31.8)
Non-medically trained provider (non-SSK)	408	63.8	(60.0–67.5)
**Source of healthcare by providers’ ownership**			
SSK provider (public)	51	8.0	(6.1–10.4)
*Medically trained providers (non-SSK)*			
Public	54	8.5	(6.5–10.9)
Private	121	18.9	(16.1–22.2)
NGO	5	0.8	(0.3–1.9)
*Non-medically trained provider (non-SSK)*			
Pharmacy	293	45.9	(42.0–49.7)
Traditional/others	115	18.0	(15.2–21.2)
**Types of care**			
Inpatient	23	3.6	(2.4–5.4)
Outpatient	616	96.4	(94.6–97.6)
**Source of care by type of patients**			
*Source of inpatient care*			
SSK scheme	10	1.6	(0.8–2.9)
Medically trained providers (non-SSK)	13	2.0	(1.2–3.5)
*Source of outpatient care*			
SSK scheme	41	6.4	(4.8–8.6)
Medically trained providers (non-SSK)	167	26.1	(22.9–29.7)
Non-medically trained provider (non-SSK)	408	63.9	(60.0–67.5)

**Fig. 2:**
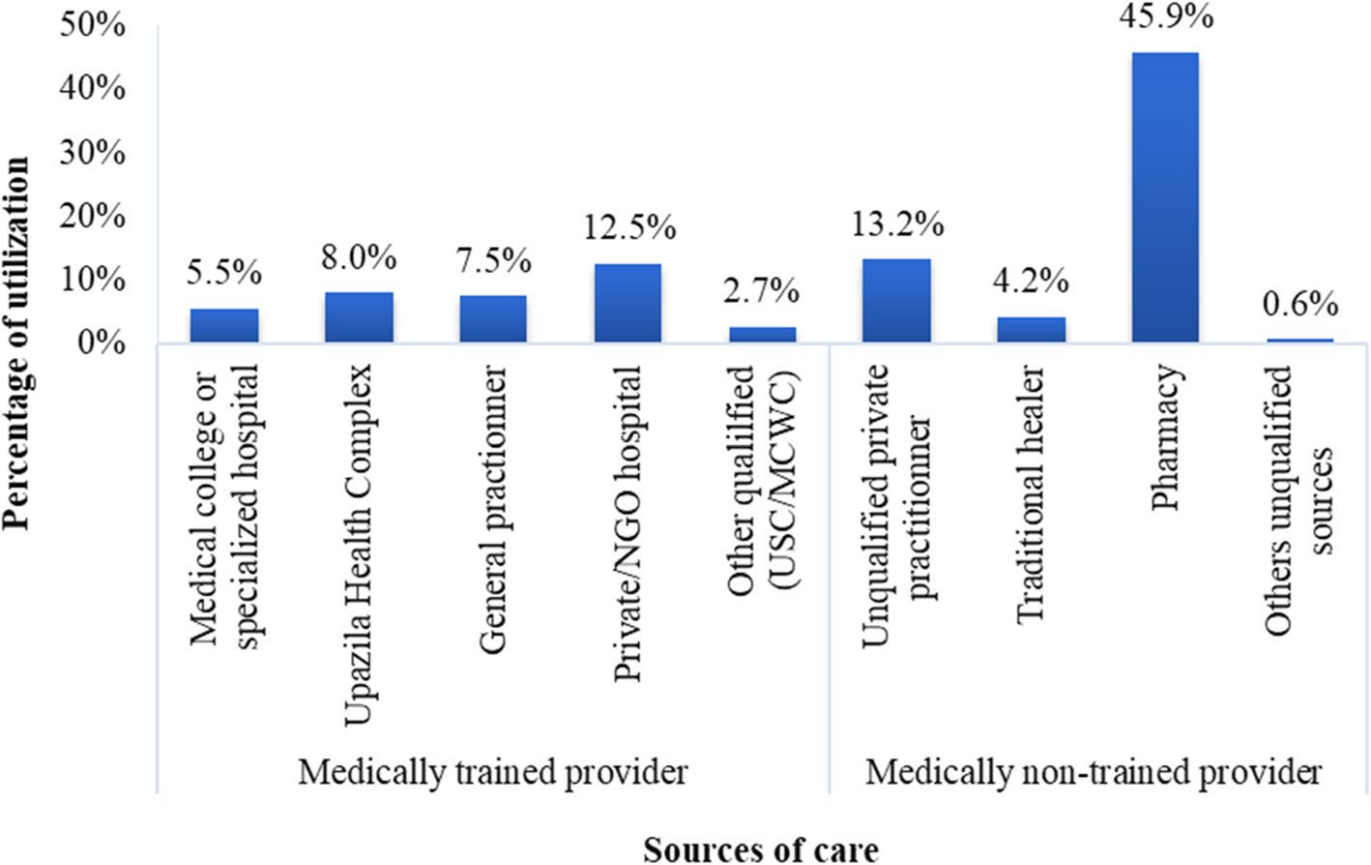
Healthcare SSK patients by sources of care

Among the patients who used MTPs, 12.5% of them went to private / non-profit hospitals followed by the SSK provider (SSK UzHC) (8%), and general practitioner (7.5%), Medical college / specialized hospitals (5.5%), and other qualified providers (USC/MCWC) (2.7%) (Fig. 2).

**Fig. 3:**
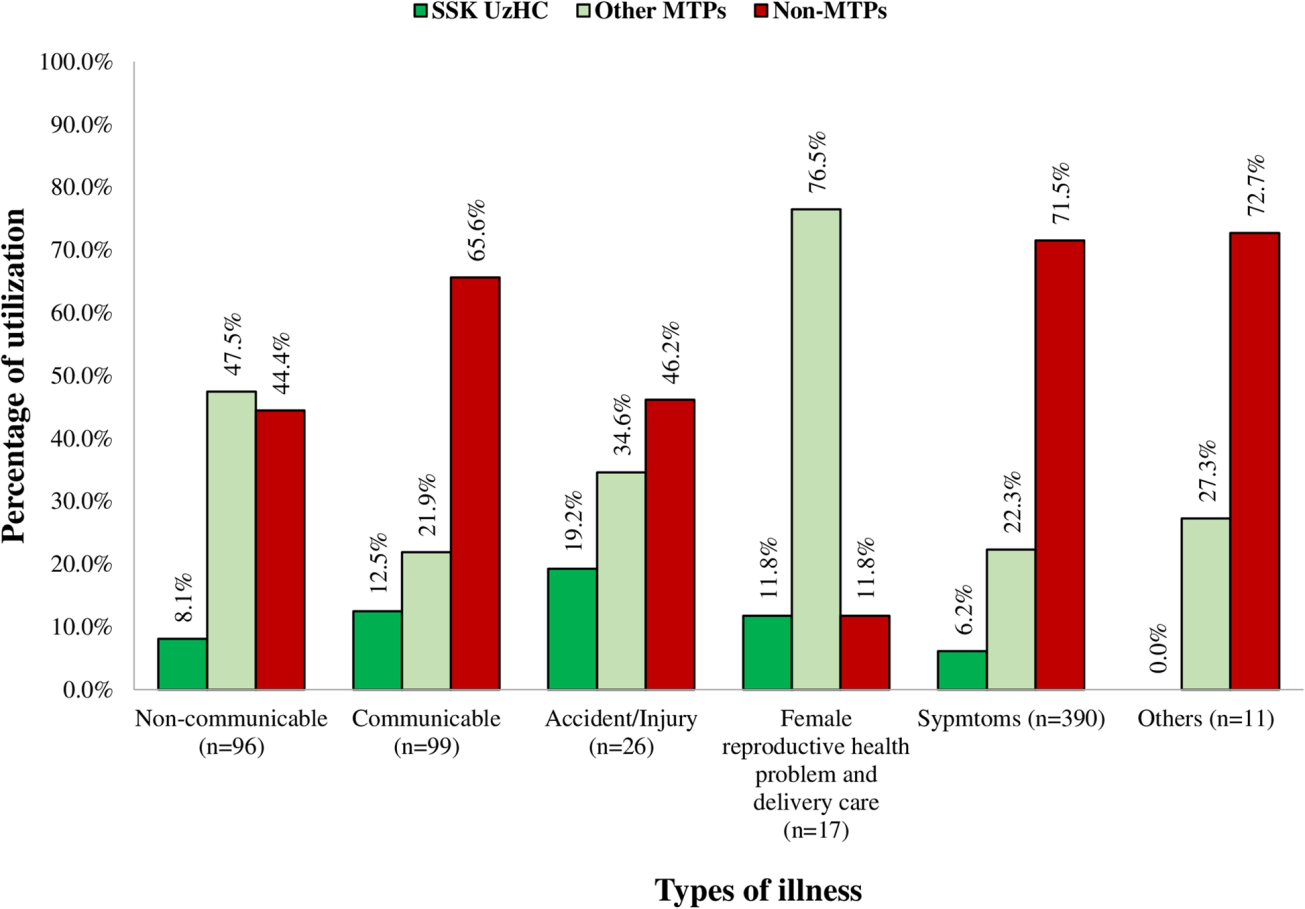
Healthcare utilization of SSK patients by type of illness and by sources of care

When comparing healthcare utilization by sources of care and types of reported illnesses, we found that 19.2% of the people with accident / injuries utilized SSK scheme, while 34.6% utilized other MTPs, and 46.2% utilized non-MTPs. Utilization of other MTPs was the highest for female reproductive health and delivery services (76.5%) and non-communicable diseases (47.5%). The utilization of non-MTP was the highest for communicable disease (65.6%), accident and injury (46.2%), Symptoms (71.5%), and other illnesses (72.7%) (Fig. 3).

### Determinants of healthcare utilization from SSK scheme

We found that the utilization of healthcare from the SSK scheme was significantly associated with education, occupation, knowledge of the scheme, distance of Unions from health facility, households’ current BPL status, monthly income, and types of self-reported illnesses (Table 5). The unemployed were about six times (OR: 5.939; 95% CI: 1.42—24.81) more likely to utilize healthcare under the SSK scheme compared to the housewives. Individuals having secondary and above level of education were less likely (0.166; 95% CI: 0.03–0.90) to utilize healthcare from SSK scheme compared the individuals having no-institutional education. If at least one member of a household heard about the SSK scheme, individuals from those households were significantly more likely to utilize healthcare from the SSK scheme (OR: 10.78; 95% CI: 2.45—47.50). We observed that currently non-BPL households were about two times more likely to utilize healthcare from the SSK scheme compared to the BPL households. Households of median and far distance Unions from the SSK UzHC were less likely to utilize healthcare from the scheme compared to the households from the nearer Unions (less than 6 km distance). We found that individuals from the second income quintile were significantly more likely to utilize healthcare from the SSK scheme compared to the poorest income quintile. Individuals from third and above income quintile were also more likely to utilize healthcare from the SSK scheme compared to the lowest income quintile but this was not statistically significant. Patients who suffered an accident and injury were almost five times more likely (OR: 4.81; 95% CI: 1.22—19.00) to utilize healthcare from the SSK scheme than those who suffered from non-communicable disease.

**Table 5:** Factors associated with the utilization of healthcare from SSK scheme and MTPs other than SSK

Socio demographic factors	Healthcare utilization from SSK scheme Adjusted OR (95% CI) ^a)^	Healthcare utilization from MTPs other than SSK Adjusted OR (95% CI) ^a)^
**Age group**		
Fewer than 18 years	**Ref**	**Ref**
18 to 34 years	3.002 (0.08,119.04)	3.195 (0.69,14.78)
35 to 44 years	1.550 (0.04,67.86)	2.362 (0.47,11.98)
45 to 60 years	1.870 (0.04,81.49)	2.217 (0.44,11.30)
> 60 years	2.805 (0.06,134.33)	2.188 (0.39,12.38)
**Sex**		
Male	**Ref**	**Ref**
Female	1.456 (0.56,3.81)	0.902 (0.52,1.57)
**Marital status**		
Married	**Ref**	**Ref**
Unmarried	0.122 (0.01,2.48)	0.448 (0.14,1.39)
Widowed/separated/destitute	0.306 (0.05,1.96)	1.153 (0.47,2.85)
Not applicable (age < = 5)	4.056 (0.08,204.21)	1.625 (0.28,9.32)
**Education of members**		
No institutional education	**Ref**	**Ref**
Primary level (years 1–5)	1.335 (0.56,3.19)	1.278 (0.77,2.12)
Secondary level and above	0.166* (0.03,0.90)	1.851* (1.05,3.25)
Not applicable (age < = 5)	1 (1.00,1.00)	1 (1.00,1.00)
**Household size**		
Less than 4 persons	**Ref**	**Ref**
4—5 persons	0.498 (0.24,1.05)	1.161 (0.77,1.74)
6 persons or more	0.909 (0.33,2.53)	0.949 (0.51,1.77)
**Occupation**		
Housewife	**Ref**	**Ref**
Student	10.89 (0.13,882.57)	2.498 (0.41,15.36)
Agricultural/labor	1.384 (0.38,5.05)	0.427* (0.21,0.87)
Service worker	1.857 (0.31,11.06)	0.762 (0.29,2.00)
Small business	1.152 (0.23,5.71)	0.526 (0.22,1.24)
Unemployed	5.939* (1.42,24.81)	0.405 (0.16,1.03)
Transport worker	0.968 (0.09,10.69)	0.099* (0.01,0.82)
Not applicable (age < = 5 years)	10.89 (0.13,882.57)	1 (1.00,1.00)
**At least one household member heard about SSK**		
No	**Ref**	**Ref**
Yes	10.78** (2.45,47.50)	0.643* (0.44,0.95)
**Current BPL status**		
BPL	**Ref**	**Ref**
non-BPL	2.275* (1.13,4.56)	1.110 (0.75,1.64)
**Distance of health facility from union**		
Nearer (less than 6 km)	**Ref**	**Ref**
Medium (7 to 15 km)	0.479* (0.23,0.99)	1.632* (1.01,2.64)
Far (More than 15 km)	0.238** (0.10,0.58)	1.267 (0.77,2.08)
**Monthly income quintile (in BDT)**		
< = 8000	**Ref**	**Ref**
> 8000 to < = 10,250	4.523* (1.27,16.11)	0.662 (0.35,1.25)
< 10,250 to < = 14,000	1.985 (0.54,7.35)	1.058 (0.60,1.87)
> 14,000 to < = 18,500	2.991 (0.82,10.86)	1.505 (0.84,2.70)
> 18,500	2.233 (0.62,7.98)	1.154 (0.64,2.07)
**Self-reported illness/symptoms**		
Non-communicable	**Ref**	**Ref**
Communicable	2.166 (0.75,6.27)	0.826 (0.44,1.56)
Accident/Injury	4.810* (1.22,19.00)	1.781 (0.71,4.46)
Female reproductive problem	2.172 (0.35,13.45)	2.596 (0.97,6.92)
Symptoms	1.156 (0.46,2.92)	0.668 (0.42,1.06)
Others (e.g., Polip, scars)	1 (1.00,1.00)	0.791 (0.20,3.11)
**Constant**	0.00162** (0.00,0.14)	0.172 (0.03,1.16)
N	766.00	781.00
Loglikelihood	-146.30	-385.60
Chi-square	82.34	72.04
*p-value*	0.000	0.000
Pseudo-R^2^	0.220	0.085

^*^ *p* < 0.05; **p < 0.01; *** *p* < 0.001

^a^ Statistical model includes the following: age, marital status, education, household size, occupation, heard about SSK scheme, current BPL status, distance from facility, monthly household income, and self-reported illness

### Determinants of healthcare utilization from other MTPs

Considering healthcare utilization from other MTPs (excluding SSK UzHC) as dependent variable, we found that individuals having secondary and above level of education were significantly more likely to utilize healthcare from other MTPs (OR: 1.85: 95% CI: 1.05–3.25) compared to the individuals having no-institutional education. Compared to the housewives, agriculture / labor and transport workers were significantly less likely to utilize healthcare from other MTPs. Interestingly, knowledge of SSK scheme were negatively associated with the utilization of healthcare form other MTPs (OR: 0.643; 95% CI: 0.44–0.95). People from medium distance Unions to the health facility was significantly more likely (OR 1.632; 95% CI: 1.01—2.64) utilize healthcare from MTPs other than SSK compared to the people from nearer distance to health facility. People from far distance Unions were also more likely to utilize other MTPs but this was not significant. Although not significant, patients with a female reproductive health problem and Accident/injury had higher odds (OR: 2.596; 95% CI: 0.97–6.92 and OR 1.781; 95% CI: 0.71—4.46) of utilizing healthcare from other MTPs compared to people with non-communicable disease. Household size, BPL status, and distance of Unions from healthcare facility had no significant association with the utilization of other MTPs.

## Discussion

Overall, 8% of the SSK members sought healthcare from the scheme of which 1.6% sought inpatient care and 6.4% sought outpatient care. The results of this study revealed that although the SSK health protection scheme provides free inpatient care and outpatient consultation services, it is yet to be popular among the enrolled BPL households, as 26.1% of the members still sought healthcare from MTPs other than the SSK scheme. However, it is still positive that out of 23 individuals requiring inpatient care, 10 of them could access healthcare services under the SSK scheme. Although utilization of the SSK scheme is low, a recent evaluation of it reported that the scheme significantly reduced the OOP expenditure and incidence of CHE among the enrolled households [30]. A similar health protection scheme in India also reported significantly increased utilization of inpatient healthcare among the beneficiaries [27]. In Vietnam, a Health Insurance scheme for the poor also substantially increased service utilization, especially for in-patient care [31]. A systematic review on the impact of health insurance in Africa and Asia found that insurance scheme improved utilization of inpatient and outpatient healthcare services [32].

One of the key reasons for such low utilization of the SSK scheme was the lack of positive knowledge about the scheme among the enrolled households. We found a significant positive association of knowledge of the SSK scheme with its utilization. Even, having knowledge on the SSK scheme is significantly negatively associated with seeking care from other MTPs. This finding of this study was similar to those of with other studies, which showed that knowledge of insurance scheme significantly affects the utilization of healthcare and dropout rates [33, 34]. Although the SSK management has developed an information, education, and communication (IEC) strategy, we found that most of the households did not have knowledge on the SSK scheme during the survey. This developed IEC strategy should be implemented appropriately among the target population and its effectiveness should be evaluated to understand on what extent the strategy could aware the members about the scheme. A study on the community-based health insurance scheme in Burkina Faso, showed that IEC campaign had positive effect on households’ knowledge about the insurance scheme [33]. Thus, SSK scheme may include strategies like delivering frequent and consistent IEC messages from multiple media channels including mobile information van, visiting member households by the programme personnel, engaging community leaders in promotional campaign of the scheme, frequent loudspeaker announcement [35], and effective outreach via health camps [36]. Furthermore, enhancing community campaigns may increase the demand for healthcare among the target population of this scheme. Another possible factor for low utilization of SSK is that most of the healthcare utilizers (390 out of 639) suffered from symptoms of illness like cough, fever, weakness, pain and discomfort, and dyspepsia. Majority of these care seekers utilized healthcare from non-MTPs (72%). They were not motivated enough to visit SSK facilities for two possible reasons. Firstly, this segment of individuals had symptoms of illness and historically they are used to utilize this kind of non-MTP providers [14]. Therefore, shifting their motivation towards utilizing MTPs will take time and may gradually improve through behaviour change intervention. Secondly, SSK scheme does not offer all types of prescribed medicines for outpatient care which they do for inpatient care. Thus, in addition to increasing awareness of the scheme among SSK members, the SSK programme can attract these non-MTP utilizers through adding benefits for outpatients to the existing benefits package i.e., ensuring all medicines for SSK outpatients. Evidence showed that involvement in insurance scheme resulted in increased utilization of outpatient care at public facilities in Ethiopia [37]. The quality of services of the SSK facilities can be improved further which may motivate the members to visit facilities and will create a positive impression in the communities. Recently, the satisfaction of inpatient services users was measured under a comprehensive evaluation of the SSK scheme [30]. The study showed that about 55% of the SSK inpatients services users were satisfied with the services they received form SSK facilities. Thus, still there is scope for quality improvement which can contribute further to increasing utilization of the scheme. Households' distance from health facility also influenced the utilization of healthcare from the scheme. Households in the Unions of medium and far distance from the health facility were less likely to utilize healthcare from the scheme. It is evident in literature that spatial distribution significantly affects healthcare utilization from health facilities [38–40]. Even it is valid for such a health protection scheme that provides free healthcare through the existing health facility.

In Bangladesh, below the UzHC level facilities, public facilities like union subcentres and community clinics provides outpatient healthcare services. On average about 36 such facilities are functional in an UzHC area [41]. The SSK scheme programme can think of including these outpatient facilities under the scheme, especially for managing outpatient care, for minimizing existing distance barriers and improving practice of visiting health facilities among the SSK members instead of going to non-MTPs. We observed that individuals from the currently non-BPL households were more likely to utilize healthcare from the SSK scheme. These non-BPL individuals may live nearer to the SSK provider and may be better informed about the SSK services. We found that better-off individuals like those belonging to the second income quintile were more likely to utilize healthcare from the SSK scheme compared to the poorest income group. And although not significant, the odds ratios were greater than one for the individuals from higher income group compared to the poorer. The stylized fact is that healthcare utilization is the lowest among the least well-off group [42].

We found that unemployed individuals were more likely to utilize healthcare services from SSK scheme. In our survey, about 41% of the unemployed individual were aged above 60 years (result not shown) and the proportion of elderly is higher in the unemployed group compared to any other occupation group. The elderly population suffers from different age-related illness and have a higher likelihood of visiting facilities for seeking healthcare. Further although not significant, the odds ratio of utilizing SSK scheme by elderly population were higher compared to the younger. It is also evident in literature that lower-income unemployed are more likely to access public health insurance and other safety net programs [43]. The use of the scheme facility was significantly higher among the people who suffered from an accident / injury compared to the people who suffered from communicable diseases. As accident and injury related problem might require immediate quality care at high costs, people were motivated to visit healthcare facility. A similar finding was observed for a study conducted in a community-based health insurance scheme in Bangladesh [28].

The SSK scheme has a potential to increase healthcare utilization among the enrolled BPL households by addressing the identified factors, namely increasing knowledge through rigorous community campaigns [44] and adding more benefits for the outpatients service to the existing benefit package of the scheme [45]. However, such a mechanism needs to be examined in terms of sustainability and moral hazard before implementation. A similar health protection scheme in India was successful in increasing utilization and improving health outcomes of the enrolled households having features of effective outreach via health camps and inclusion of private facilities for providing healthcare [36] which can also be considered for SSK scheme in Bangladesh. Furthermore, the list of BPL households should be updated regularly for precise targeting and providing services to those who cannot afford it. Such precision may be possible by increasing local-level political commitment. Since healthcare utilization had a significantly negative association with the distance from the SSK facility, the authority can think of linking the lower-level facilities such as union sub-center where the patient will receive outpatient care and be referred to higher-level facilities for inpatient or complicated cases. However, further exploration would be required before implementing such an initiative.

### Limitations

The study had a few limitations. Firstly, the sample households were selected using a simple random sampling technique; however, during the survey, we found that some of the selected households were not eligible to receive SSK services as they did not have SSK cards. So, we interviewed the adjacent households instead of the selected households, and this can be one possible shortcoming of this study. The interview of such adjacent households may create selection bias. However, we used a 3% error level margin to capture more households in the sample to reduce such bias. This was the first study that examined the healthcare utilization and knowledge of the SSK scheme and associated factors among the BPL households of SSK scheme in Bangladesh. Another limitation of this study was that the seasonal variation in utilization of healthcare had not been captured. This is because the survey was carried out from July to September 2018, which restricted establishing the causality in a vigorous way. Although we used the distance variable in the regression model as determining factor, some other important variables (e.g., travel time and cost) were not considered as these can vary according to the type of road and the transportation facility. We have used 90 days recall period for capturing both inpatient and outpatients in the households which resulted in reduced number of inpatients in the sample. Thus, the results should be carefully interpreted considering this limitation. Furthermore, as data were collected through self-reported questionnaire, there is a possibility of recall bias while reporting illness and healthcare. Earlier studies used recall periods of 1 to 12 months to collect similar data [46–48]. We used a 90-day recall period to reduce the possibility of such recall bias.

## Conclusions

This study investigated healthcare utilization behaviour of the financially disadvantaged BPL population after including them in a health protection scheme. Utilization of healthcare from the scheme facility is low despite the provision of free inpatient care for 78 different types of diseases and outpatient consultation. Knowledge of the scheme was one of the critical barriers to the utilization of the SSK scheme. With appropriate measures in place, such as developing and implementing interpersonal communication strategies and organizing regular awareness-building campaigns, and promotional activities at the community level, the knowledge of the enrolled population may increase over time. Furthermore, as many people utilized care from non-qualified and other MTPs, the policymakers may think of redesigning the benefit package based on the needs of the BPL population which may increase the demand for healthcare from the scheme among them. The evidence generated from this study will help to address the reasons behind the low utilization and the challenges to increase the effectiveness of SSK in improving the healthcare seeking behaviour of the BPL population. Addressing the drawback associated with healthcare utilization is essential before further expansion or scale-up of the scheme. A successful implementation and scale-up of this scheme will be a vehicle to achieve UHC by addressing the healthcare needs of the people who cannot afford quality healthcare in Bangladesh.

## Supplementary Information

**Additional file 1.**

**Additional file 2.**

**Additional file 3.**

## Data Availability

All data generated or analysed during this study are included in this published article (and its Supplementary information files 2 and 3).

## Abbreviations

BPL: Below Poverty Line
CBHI: Community Based Health Insurance
CHE: Catastrophic Healthcare Expenditure
CI: Confidence Interval
GoB: Government of Bangladesh
HEU: Health Economics Unit
IEC: Information, Education, And Communication
LMICs: Low- and Middle-Income Countries
MCWC: Maternal and Child Welfare Centre
MoHFW: Ministry of Health and Family Welfare
MTPs: Medically Trained Providers
NGO: Non-Government Organization
OOP: Out-Of-Pocket
RSBY: Rashtriya Swasthya Bima Yojana
SDGs: Sustainable Development Goals
SSK: Shasthyo Suroksha Karmasuchi
UHC: Universal Health Coverage
UzHC: Upazila Health Complex
USC: Union Sub-Centre
