# Supplementary material for: Factors affecting the healthcare utilization from Shasthyo Suroksha Karmasuchi scheme among the below-poverty-line population in one subdistrict in Bangladesh: a cross sectional study
Source: BMC Health Serv Res. 2022 Jul 8;22:885. doi: 10.1186/s12913-022-08254-1 (PMC9270808; doi:10.1186/s12913-022-08254-1)
Supplement: Supplementary file 1 — Additional file 1. [file 12913_2022_8254_MOESM1_ESM.pdf]

Household survey questionnaire (English)

Study title: A study to systematically document the implementation related challenges of *Shasthyo Suroksha Karmasuchi* Health Protection Scheme of the Government of Bangladesh

Principal Investigator PI: Sayem Ahmed
Organization: icddr,b

**Section 1: Identification**
**Questionnaire code:**
**Name of the respondent** ____<br>Is the respondent a household head? ......1. Yes ...... 2. No<br>Address of the respondents-For SSK members write as mentioned in the card: ____<br>____<br>Union: |___|___| Code-1<br>Mobile number: |___|___|___|___|___|___|___|___|___|___|___| **Phone number: Yours 1 Not yours 2 If others, Please mention the relationship with the person** ____________________
Is the household under SSK services? ☐ 1. Yes ☐ 2. No
Code of the interviewer: |___|___| Interview starting time: |___|___|: |___|___| ☐ am/ ☐ pm<br>Interview end time: |___|___|: |___|___| ☐ am/ ☐ pm<br>Date of the interview: |___|___|:|___|___|:|___|___|___|___| Day/Months/Year<br>Record GPS location:

Code-1

1.Kokodhora
2.Gohaliabari
3.Doshkia
4.Durgapur
5.Nagbari
6.Birbasinda
7.Sohodebpur
8.Narandia
9.Paikora
10.Parokhi
11.Bolla
12. Bangra
13.Solla
14.Alenga municipality

Version date: 22.03.18

Section 2: Information on household members

C1	C2	C3	C4	C5	C6	C7	C8	C9	C10	11 C11
Member code	Name Short name	Relationship with the household head	Gender	Age-in integars	Marital status	Educational qualification of the member in years If doesn't have education write "0"	Main occupation	Does this member earn regularly? Yes 1 No 2 Not-appilcable 99]	How many days the memebr worked in the last 30 days	Earnings of the last 30 days [In taka]
		1 Self<br>2 Husband/wife<br>3 Father/Mother<br>4 Son/daughter<br>5 Father-in-law / mother-in-law<br>6 Brother/Sister<br>7 Son-in-law / daughter-in-law<br>8 Grandson/Grand- daughter<br>9 Relatives<br>10 Adopted children<br>11 Uncle/aunt<br>12 Grandfather/grandmother<br>13 Relation with who are not relatives<br>14 Others Specify	1 Male<br>2 Female		1 Married<br>2 Single<br>3 Divoreced/divorcee<br>4 Separated<br>5 Destitute<br>6 Others-Please specify		1 Agriculture<br>2 Housewife<br>3 Daylaborer<br>4 Rishkaw/Van driver<br>5 CNG/Auto driver<br>6 Maid<br>7 Fromal Job<br>8 Shopkeeper<br>9 Begger<br>10 Unemployed<br>11 Student<br>12 old<br>13 Child<br>14 Farming<br>15 Sharecropping<br>16 Worker<br>17 Fisherman/oarsman/Fish farming<br>18 Handycraft<br>19 Garments worker<br>20 Reastauratn/worker<br>21 Mechanics<br>22 Salesman<br>23 Barber<br>24 Tailor<br>25 Teacher<br>26 Mobile business<br>27 Not applicable<br>28 Disabled<br>29 Others-specify			

Section 3: Information on household

Part 3A: Living place and land related information

1	Are you the owner of the homesteadyou used to live?	1. Yes → go question no 3	2. No	
2	Who is the owner of the homestead?	1. Others land<br>2. Roadside/govt. land<br>3. Rented<br>(Please mention the area if it is rented) -----sq.ft		
3	How was the living condition of the household? According to the interviwer	1. Clean<br>2. Average<br>3. Dirty<br>4. Very dirty		
4	Do you have any other land except the homestead?	1.Yes	2. No	
5	Write the amount of land/homestead area	Homestead with pond and pool area:<br>Paki :_______Decimal:_______ = Decimal|___|___|___|___| Agricultural land<br>Paki:_______ Decimal: _______ = Decimal|___|___|___|___|		
6	How many rooms are there in this household?	Number |___|		
7	What kind of materials was used to make the main room of the household to be verified?	1. Pucca / semi-Pucca<br>2. Tin<br>3. Tin and bamboo<br>4. Tin and others<br>5. Bamboo and others<br>6. Soil<br>7. wood<br>8. ceramics/ tiles<br>9. Others		

	Roof	Wall	Floor
Serial no			

8 Are these goods / assets available at your household? (More than one answer is acceptable?)

No.	goods / assets	Yes	No
1.	Cow/goat	1	2
2.	Fishing net	1	2
3.	duck/Chicken	1	2
4.	Grocery	1	2
5.	Riksha/Van/ Auto Riksha	1	2
6.	Agticultural instruments	1	2
7.	Boat	1	2
8.	Bed	1	2
9.	blanket	1	2
10.	Quilt	1	2
11.	Hurricane	1	2
12.	Chair/ Table	1	2
13.	Dining Table	1	2
14.	Amirah / Showcase	1	2
15.	Sofa Set	1	2
16.	Television	1	2
17.	Radio/ Tap Recorder	1	2
18.	Clock/wall clock	1	2
19.	Telephone/ Mobile Phone	1	2
20.	Bicycle	1	2
21.	Motorcycle	1	2
22.	Freeze	1	2
23.	Fan	1	2
24.	Sewing-machine	1	2
25.	OthersSpecify______________________	1	2

9 Which type of toilet do you use?

No.	Toilet type	Yes	No
1	Pit Toilet	1	2
2	Paka Toilet	1	2
3	Soil made Toilet	1	2
4	Sanitary	1	2
5	Others Specify]_____________________	1	2

10 What is the main source of drinking water?

No.	Source type		
1	Tube well	1	2
2	Ponds/rivers/puddles/streams	1	2
3	Rain Water	1	2
4	Pipe/tap/Supply Water	1	2
5	Pure/filtered water	1	2
6	Others please specify_____________________	1	2

11	Do the household have Electricity?	1. Yes	2. No		

Part 3B: **Other sources of household income**						
12	Do the household have any permanent income source?	1. Yes	2. No			
14	Do the household has any child labor?	1. Yes	2. No	Not appilicable 99		
15	Section-2<br>In the last 12 months, what were the other sources of household irregular income? Exclude the items considered in the section-2	**No.**	Sources of income		Amount of income in Taka	
		1.	Food for work			
		2.	Income from assets			
		3.	VGD/destitute allowance			
		4.	Old allowance			
		5.	Pension			
		6.	Freedom fighter allowance			
		7.	Rremittence			
		8.	Stipend/scholarship			
		9.	Other allowances			
		10.	Others sources specify_____________			

Part 3C: **Loan information of Household**

16	In the last 12 months, was any household member involved with loan?		1. Yes	2. No		Code: 2
	If yes please fill-up the following section					
	Member code	What is the source of loan?<br>1. Community scheme<br>2Small lender organization<br>3From Bank<br>4. Other Specify].......	How much money did you loan in the last time?	What is the left-over ammount of your loan?	Where did you invest the borrowed money during the last year? Code: 2	

1. Poultry farming	10. To buy fishing equipment
2. Cow/goat farming	11. Buy boat
3. Handy crafting	12. Buy van /rickshaw
4. Agriculture	13. Small business
5. House building/repairing	14. Business
6. Marriage	15. Buy agricultural equipment
7. To buy consumption goods	16. Pay the borrowed amount
8. For educational expenses	17. Sent abroad
9. For treatment	18. Others

Part 3D: **Household Food Security related information**				
18	How often did you eat three `square meals' full stomach meals a day in the past 12 months not a festival day?	1 Mostly 3 meals each day<br>2. Sometimes 3 meals per day<br>3. Rarely 3 meals per day 1-6 times this year<br>4. Never		
19	In the last 12 months how often did you yourself skip entire meals because there was not enough food?	1. Never<br>2. Rarely 1-6 times this year<br>3. Sometimes 7-12 times this year<br>4. Often few times each month		
20	In the last 12 months how often did you personally eat less food in a meal because there was not enough food?	1. Never<br>2. Rarely 1-6 times this year<br>3. Sometimes 7-12 times this year<br>4. Often few times each month		
21	In the last 12 months, how often did you or any of your family have to eat wheat or another grain although you wanted to eat rice not including when you were sick?	1. Never<br>2. Rarely 1-6 times this year<br>3. Sometimes 7-12 times this year<br>4. Often few times each month		
22	In the past 12 months how often did your family have to ask food from relatives or neighbors to make a meal?	1. Never<br>2. Rarely 1-6 times this year<br>3. Sometimes 7-12 times this year<br>4. Often few times each month		
23	How many months you can live on from your land income?	______Month		

Section 4:Household expenditure related information

C1	C2	C3
Sl. no	Types of food	Amount consumed If do not consumed put "0"
**Part A: Food Weekly expenditure**		
**4.1 Rice crop consumed in the last week**		
1	Rice coarse (Kg)	
2	Rice-plain (Kg)	
3	Wheat (Kg)	
4	Flour (Kg)	
5	Flattened/Puffed rice（Kg)	
**4.2 Pulse crop consumed in the last week**		
1	Lentil (kg)	
2	Bengal gram pulses (Kg)	
3	Mush Kalai (Kg)	
4	Pea gram (Kg)	
5	Chick ling-Vetch (Kg)	
6	Green gram (Kg)	
7	Anchor (Kg)	
**4.3 cooking oil consumed in the last week**		
1	Soybean oil (liter)	
2	Mustard oil (liter)	

3	Dalda (liter)	
4	Ghee (Kg)	
5	Palm Oil (litter)	
6	Others Specify________	
**4.4 leaf vegetables consumed in the last week**		
1	Amaranth (Kg/bundle)	
2	Malabar spinach (Kg/bundle)	
3	Spinach (Kg/bundle)	
4	Spinach ipomoea aquatic (Kg/bundle)	
5	Others leafy (Kg/bundle)	
6	Others leafy （Kg/bundle）	
7	Others leafy （Kg/bundle）	
8	Potato （Kg）	
9	Pointed gourd （Kg）	
10	Bitter melon （Kg）	
11	Cauliflower （Kg/piece）	
12	Cabbage （Kg/Piece）	
13	Brinja (Kg)	
14	Arum (Kg)	
15	Gourd (Number)	
16	Tommato (Kg)	
17	Green papaya (Kg）	
18	Green Banana (Number)	
19	Green Chili (Kg)	
20	Onion (Kg)	
21	Garlic (kg)	
22	Ginger (kg)	
23	______Others vegetables (Kg)	
24	_____Others vegetables (Kg)	
25	______Others vegetables (Kg)	
**Part B: Food Monthly consumption]**		
**4.5** Spices consumption in the last month		
Sl. no	Types of food	Amount consumed (If do not consumed put "0")
C1	C2	C3
1	Dried chili（gm）	
2	Turmeric (gm）	
3	Cumin (gm)	
4	Cinnamon (gm）	
5	Cardamom (gm)	
6	Others spice_____gm	
7	Others spice______ gm	
8	Others spice______ gm	
**4.6 Fish, meat, egg and milk consumption in the last month**		
1	Large Fish (kg)	
2	Smaller Fish kg]	
3	Dried fish (kg)	
4	Beef (kg)	
5	Buffalo meat (Kg)	
6	Local chicken meat (kg)	
7	Poultry chicken meat (Kg)	
8	[Duck meat (Kg)	
9	Mutton (Kg)	
10	Egg (Number)	
11	Milk (Litter)	
12	Other protein items__ __（Kg)	
13	Other protein items__ __（Kg)	
14	Other protein items__ __ Kg	
**4.7 [Fruits consumption in the last months]**		
1	Banana (Number)	
2	Chinese (date	
3	Olive (Kg)	
4	Papyaa (Kg)	
5	Mango (Kg)	
6	Jackfruit (Number)	
7	Java Plum (Kg)	
8	Apple (Kg)	
9	Orange (Number)	
10	Graps (Kg)	

11	Guava (Kg)	
12	Pineaple (Number)	
13	Other fruits _________ Kg	
14	Other fruits _________ Kg	
15	Other fruits _________ Kg	
**4.8 Other foods in the last month**		
1	Sugar (Kg)	
2	Jaggery (Kg)	
3	MIlk powder (Kg)	
4	Salt (Kg)	
5	Noodles (Packate)	
6	Semolina (Kg)	
7	Cold drink (litter)	
8	Sweetned food	
9	__________________ Child food (Kg)	
10	Tea leaf (Kg)	
11	Biscut (Pack)	
12	Bombay mix (Packet)	
13	Others_________ Number	
14	Others_________ Number	
15	Others_________ Number	
**Part C:** Tobacco products Weekly expense		
**4.9** Tobacco intact in last week		
1	Batel leaf（number）	
2	Betel nut (number)	
3	Tobbacco leaf (Kouta)	
4	Tobbacco (number)	
5	Gul (Kouta)	
6	Biri (Packet)	
7	Cigaret (Packet)	
8	Others__________________ Number	
9	Others__________________ Number	
10	Others__________________ Number	

**Part D: [Non-food Monthly expenditure]**		
**C1**	**C2**	**C3**
**Sl. no**	**Types**	**Expenditure ammount Monthly**
**4.10** Non food expenditure in last month		
1	House rent	
2	Electricity Bill/Solar Power	
3	Gas bill	
4	Fuel wood	
5	Fuel oil (kerosene, petrol)	
6	Transport	
7	Health	
8	Education school fees, (coaching, private teacher, etc.)	
9	Notebooks/Pens/books/other study materials]	
10	Communication expense (mobile, telephone, etc.)	
11	Dish bills/internet bill	
12	Cosmetics expense (Cream, Powder, make-up etc.)	
13	Hair Oil/Coconut Oil	
14	Cleaning and hygiene (soap, detergent, shampoo etc.)	
15	Laundry	
16	Co-operative instalment/ Micro-credit instalment	
17	Salon/Spa	
18	Others Specify	

Part E: [Non-food Yearly expenditure]		
**4.11 Non-food expenditure in last year**		
**Sl.no**	**Types**	**Expenditure amount in the last year**
1	Clothing	
2	Shoe	
3	Quilt/quilt/pillow/blanket/bed	
4	Entertainment/religious festival	
5	House construction/House Renovation	
6	Furniture	
7	Insurance Premium	
8	House keeper/ maids salary	
9	Gifts provide	
10	Donation	
11	Travel	
12	Others Specify	

Section 5: Treatment and expenditure related information of the household members in the last three months (Write individual information in separate row)

Does any household member got sick or have symptoms of sickness in the last three months? 1. Yes 2. No

1	2	3	4	5	6		7		8	9										10	11	12	13	14					
Member code	The disease code of the member	How many days you/ the member suffered from illness?	Did you or the member sought treatment for that disease?<br>Code: Yes1 No2 If answer is no Only question 5 is applicable	If you didn't seek treatment, why?<br>1. Problem was not severe<br>2. The cost for treatment was very high<br>3. Didn't have enough money<br>4. Long distance of the health facility<br>5. No one was available to take to the facility<br>6. Didn't know the location of facility<br>7. [If others, specify]	Where did you seek treatment from?<br>1. Medical College Hospital<br>2. Specialized hospital<br>3. [Referral district hospital]<br>4. Other District Hospital<br>5. Upazila Health Complex<br>6.Union Sub center/CC/Rural dispensary<br>7. Maternal and Child Welfare Center<br>8. Private Clinic and Hospital<br>9. NGO Clinic/ Hospital<br>10. NGO Health Worker<br>11. Government Health Worker<br>12. Qualified Private Practitionar<br>13. Unqalified Private Practitionar<br>14. Ayurvedic /homeopathic<br>15. Self-treatment<br>16. Pharmacy<br>17. OthersSpecify...]		[Did you get admitted to the hospital for that problem? Code: Yes1 No 2; If no then go the question no 9]	If yes, how many days you were admitted to the hospital? in full days]	[Was your treatment covered by the SSK?]	[Cost for treatment If any item is not applicable, palce "0"]										[How many days did you absent from your work due to illness? Full days]	[In the last three months, despite of illness how many days were you present at your work? write "99" if not applicable]	[In the last three months, did you left any work permanently due to illness? yes1, No 2, Not apicable 99]	In the last 12 months, did any of the household member had to leave school permanently? Yes 1 No 2 Not applicable 3	How did you manage the treatment cost?<br>1. Regular income<br>2. Family savings<br>3. SSK provided<br>4. Insurance<br>5. Selling of own materials<br>6. Selling of Trees/Agricultural crops/domestic animals<br>7. Selling of permanent property<br>8. Mortgage of land or property<br>9. Borrowing money from lender<br>10. Help from friends or relatives<br>11. Borrow from Relatives/friends/colleagues<br>12. Loan from institute/association<br>13. Others, Please specify					
					Sl. no	Source of treatment				Consultai	Medicine	Bed rent	Diagnostic	Transport	Tips	Attednant	Package	Operation	Others					উৎস-1	টা	উৎস-2	টা	উৎস-3	টা
					1.																								
					2.																								
					3.																								
					1.																								
					2.																								
					3.																								
					2.																								
					3.																								

* 1. Cough 2. Fever 3. Malaria 4. Typhoid 5. Diarrhoea/dysentery 6. Vomiting 7. Asthma/breathlessness 8. Minor injury 9. Bone fracture 10. Jaundice 11. Weakness 12. Worms 13. Pain/discomfort 14. Skin disease 15. Convulsion/Seizure 16. Dyspepsia 17. Hypertension 18. Diabetes 19. Tuberculosis 20. Leprosy 21. Injury/Paralysis 22. Arthritis 23. Rheumatic Fever 24. Cancer 25. Delivery 26. Obstetric emergency 27. Mental problem 28. Others

**Section6: Effect of illness on the food security**<br>If no member of the household suffers from any illness, start from the next section		
**Sl.no**	**Question**	**Type of Answer**
1	In the last three months, did your family have to squeeze consumption expenditure due to the illness of any member?	□ 1. Yes<br>□ 2. No<br>□ 99. Not applicable
2	In the last three months, did your family have to squeeze other expenditure e.g., education, clothing, furniture due to the illness of any member?	□ 1. Yes<br>□ 2. No<br>□ 99. Not applicable

Section 7: Effect of Health expenditure and SSK related Question

Sl.no 1	Question 2	Type of answer 3	
1	Do you think that many people faces financial catastrophe while seeking healthcare service?	□ 1. Yes □ 2. No	
2	Do you think, some of your neighbour used to work despite of their illness?	□ 1. Yes □ 2. No	
3	Do you know, different institutions used to bear treatment cost of its members?	□ 1. Yes □ 2. No	
4	Have you ever heard of life insurance?	□ 1. Yes □ 2. No	
5	Have you ever heard of health insurance?	□ 1. Yes □ 2. No	
6	Do you know, there is a health protection scheme in this area for the poor people?	□ 1. Yes □ 2. No	
7	Do you know, how SSK services are provided from the healthcare facility?	1. By card<br>2. By Cash<br>3. Don't know	
8	Do you know, how many disease are covered under SSK scheme?	□ 1. Yes<br>□ 2._____ No （If yes, ask how many?）	
9	What types of treatment expenses are borne by the SSK?Multiple responses are acceptable	1. Free medicine<br>2. Free diagnostic/test<br>3. Referral facility<br>4. Provide expense for referral to District hospital	
10	Where did you learn about the SSK?	1. From others in your locality<br>2. Representative from SSK<br>3. From familiar person<br>4. From local leader<br>5. From Miking<br>6. From poster<br>7. From billboard<br>8. From Television commercial<br>9. Others, please specify	
**Part 7A: SSK service related information**			
11	Card number of SSKFill from the card	|___|___||___|___||___|___||___|___||___|___||___|___||___|___||___|___|___|	
12	Card accepting date	|___|___|:|___|___|:|___|___|___|___| Day/Month/Year	
13	Write the code of card issued person from **Section 2**	|___|___| Member code	
14	Did you ever use SSK card? if didn't why?	1. Yes	2. No (go to the next section)
15	How many times did you use the card?	___________ times	

Section 8: Recent Health related question

In the below table, Each group has three boxes, put tick marks only in one of the boxes that indicate your physical condition.

Code:											
1	**walking**<br>1) I have no problem in walking<br>3) I have problem in walking<br>3) Cannot move from bed										
2	**Self-care**<br>1) I have no problem with self-care<br>2) I have problems washing or dressing myself<br>3) I am unable to wash or dress myself										
3	**Usual activities**<br>1) I have no problem with performing my usual activities<br>2) I have some problems with performing my usual activities<br>3) I am unable to perform my usual activities										
4	**Pain/ Discomfort**<br>1) I have no pain or discomfort<br>2) I have moderate pain or discomfort<br>3) I am extreme pain or discomfort										
5	**Anxiety/ Depression**<br>1) I am not anxious or depressed<br>2) I am moderately anxious or depressed<br>3) I am extremely anxious or depressed										
6<br>**Indicate todays physical condition in this scale**<br>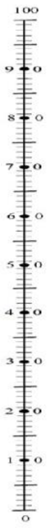	To help people say how good or bad a health state is, we have drawn a scale (rather like a thermometer) on which the best state you can imagine is marked 100 and the worst state you can imagine is marked 0. We would like to indicate on this scale how good or bad your own health is today, in your opinion. Please do this by drawing a line from the box below to whichever point on the scale indicates how good or bad your health state is today.<br>Your own health state today										

Thank You

Household survey questionnaire (Bengali)

Section 1: পরিচিতি সংক্রান্ত তথ্য
**Questionnaire code:**
উত্তরদাতার নামঃ ____<br>উত্তরদাতা কি থানাপ্রধান …… 1. হ্যাঁ 2. না<br>উত্তরদাতার ঠিকানা : ____<br>____<br>ইউনিয়নঃ |___|___| (কোড-1)<br>ফোন নাম্বারঃ |___|___|___|___|___|___|___|___|___|___|___| ফোন নাম্বারঃ নিজের (1) অন্যের (2) (অন্যের হলে সম্পর্ক উল্লেখ করুন ____________________________)
থানা কি এসএসকে সেবাভুক্ত 1. হ্যাঁ 2. না
সাক্ষাৎকার গ্রহণকারীর কোডঃ |___|___| সাক্ষাৎকার গ্রহণ শুরুর সময়ঃ|___|___|: |___|___| ☐ am/ ☐ pm<br>সাক্ষাৎকার গ্রহণ শেষের সময়ঃ|___|___|: |___|___| ☐ am/ ☐ pm<br>সাক্ষাৎকার গ্রহণের তারিখঃ|___|___|:|___|___|:|___|___|___|___|(দিন/মাস/বছর)<br>জিপিএস লোকেশনঃ

কোড-1

1. কোকডহড়া
2. গোহালিয়াবাড়ী
3. দশকিয়া
4. দুর্গাপুর
5. নাগবাড়ী
6. বীরবাসিন্দা
7. সহদেবপুর
8. নারান্দিয়া
9. পাইকড়া
10. পারখি
11. বল্লা
12. বাংড়া
13. সল্লা
14. এলেঙ্গা পৌরসভা
15. কালিহাতি

Section 2: খানার সদস্যদের তথ্য

ক1	ক2	ক3	ক4	ক5	ক6	ক7	ক8	ক9	ক10	ক11
(সদস্যকোড)	নাম<br>(সংশ্লিষ্ট নাম লিখুন)	থানা প্রধানের সাথে সম্পর্ক<br>1 থানাপ্রধান নিজে<br>2 স্বামী/স্ত্রী<br>3 বাবা/মা<br>4 পুত্র/কন্যা<br>5 শ্বশুর/শাশুড়ি<br>6 ভাই/বোন<br>7 জামাতা/পুত্রবধূ<br>8 নাতি/নাতনী<br>9 আত্মীয়<br>10 পালিত সন্তান/সৎ সন্তান<br>11 চাচী/মামি/খালা/ফুফু<br>12 দাদা/দাদী<br>13 আত্মীয় স্বজন নয় এমন<br>14 অন্যান্য (উল্লেখ করুন)	লিঙ্গ<br>1 পুরুষ<br>2 মহিলা	বয়স<br>(পূর্ণবছর)	বৈবাহিক অবস্থা<br>1 বিবাহিত<br>2 অবিবাহিত<br>3 বিপত্নীক/বিধবা<br>4 তালাকপ্রাপ্ত/পৃথকীকৃত (Separated)<br>5 স্বামী পরিত্যক্তা<br>6 অন্যান্য (উল্লেখ করুন)	সদস্য কত বছর প্রাতিষ্ঠানিক শিক্ষাগ্রহন করেছে (শিক্ষা না থাকলে "0" লিখুন)	প্রধান পেশা<br>1 কৃষিকাজ; 16 শ্রমিক<br>2 গৃহিণী; 17 জেলে/মাঝি/মৎসচাষী<br>3 দিনমজুর; 18 ক্ষুদ্র কুঠিরশিল্প<br>4 রিক্সা/ভ্যান চালক; 19 গার্মেন্টস কর্মী<br>5 চালক (অটো/সিএনজি); 20 রেস্টুরেন্ট কর্মী<br>6 গৃহকর্মী; 21 মিস্ত্রী/ইলেক্ট্রিশিয়ান<br>7 চাকুরী; 22 দোকান কর্মচারী<br>8 দোকানদার; 23 নাপিত<br>9 ভিক্ষাবৃত্তি; 24 দর্জি<br>10 বেকার; 25 শিক্ষক<br>11 ছাত্রছাত্রী; 26 ভ্রাম্যমাণ ব্যাবসা<br>12 বৃদ্ধ/বৃদ্ধা; 27 প্রযোজ্য নয়<br>13 শিশু; 28 শারীরিক ভাবে অক্ষম<br>14 খামার (কৃষি, মৎস, দুগ্ধ ও মুরগী); 29 অন্যান্য (উল্লেখকরুন)<br>15 বর্গাচাষ	এই সদস্যের কি নিয়মিত উপার্জন হয়? হ্যাঁ (1) না (2) প্রোজোয্য নয় (99)	গত ৩০ দিনে কতদিন কাজ করেছেন	গত ৩০ দিনে আয় (টাকায়)

Version date: 29.05.18

Section 3: খানা সংক্রান্ত তথ্য

Part 3A:বসতভিটা ও জমাজমি সম্পর্কিত তথ্য

1	আপনারা যে বাড়িতে থাকেন তার জমির মালিক কি আপনারা নিজে?	1. হ্যাঁ (→ 3 নং এ যান)	2. না
2	বাড়ির জমি নিজের না হলে কার?	1. অন্যের জমি<br>2. রাস্তায়/সরকারী কোন জমি<br>3. ভাড়া করা<br>ভাড়া করা হলে আয়তন লিখুন ------বর্গফুট	
3	সাক্ষাৎকার গ্রহনকারীর দৃষ্টিতে খানার আশেপাশের পরিবেশ কেমন? (নিজে পর্যবেক্ষণ করবেন)	5. পরিস্কার<br>6. মোটামুটি পরিস্কার<br>7. নোংড়া<br>8. খুব নোংড়া	
4	বাড়ির জমি ছাড়া আপনাদের পরিবারের অন্য কোন জমি আছে কি?	1. হ্যাঁ	2. না
5	পরিবারের জমি/বসত ভিটার পরিমান লিখুন	বসত ভিটা (পুকুর এবং ডোবা সহ বাড়ির জমি):<br>পাকিঃ_______শতাংশ_______= শতাংশ |___|___|___|___| কৃষি জমিঃ<br>পাকিঃ_______শতাংশ_______= শতাংশ |___|___|___|___|	
6	খানায় কতগুলি ঘর আছে?	সংখ্যাঃ |___|	
7	প্রধান ঘর কি কি উপকরণ দ্বারা তৈরী (যাচাই করুন)	10. পাকা/ আধা পাকা<br>11. টিন<br>12. টিন এবং বাঁশ<br>13. টিন এবং অন্যান্য<br>14. বাঁশ এবং অন্যান্য<br>15. কাঁচা (মাটি)<br>16. কাঠ<br>17. সিরামিক/টাইলস<br>18. অন্যান্য	ছাদঃ |___| দেওয়ালঃ |___| মেঝেঃ |___| অন্যান্য হলে উল্লেখ করুনঃ<br>ছাদ___________<br>দেওয়াল________<br>মেঝে__________

8 উল্লেখিত দ্রব্যসামগ্রী/ সম্পদগুলি কি আপনার খানায় আছে? (একাধিক উত্তর হতে পারে)

নং	পণ্য/ দব্যসামগ্রী	হ্যাঁ	না
26.	গরু/ ছাগল	1	2
27.	মাছ ধরার জাল	1	2
28.	হাঁস / মুরগী	1	2
29.	মুদি দোকান	1	2
30.	রিক্সা / ভ্যান / অটো রিক্সা	1	2
31.	কৃষি যন্ত্রপাতি (সেচ মেশিন, চাষকরার যন্ত্র)	1	2
32.	নৌকা	1	2
33.	খাট / চৌকি	1	2
34.	কাথা / কম্বল	1	2
35.	তোষক	1	2
36.	হারিকেন	1	2
37.	চেয়ার / টেবিল	1	2
38.	ডাইনিং টেবিল	1	2
39.	আলমারি / শোকেস	1	2
40.	শোফা সেট	1	2
41.	টেলিভিশন	1	2
42.	রেডিও / টেপ রেকর্ডার	1	2
43.	ঘড়ি / দেওয়াল ঘড়ি	1	2
44.	টেলিফোন / মোবাইল ফোন	1	2
45.	বাইসাইকেল	1	2
46.	মোটর সাইকেল	1	2
47.	ফ্রিজ	1	2
48.	ফ্যান	1	2
49.	সেলাই মেশিন	1	2
50.	অন্যান্য (উল্লেখ করুন)________________	1	2

9 আপনার কোন ধরনের পায়খানা ব্যবহার করেন?

নং	পায়খানার ধরন	হ্যাঁ	না
1	স্লাব পায়খানা	1	2
2	পাকা পায়খানা	1	2
3	কাঁচা পায়খানা	1	2
4	সেনিটারী	1	2
5	অন্যান্য (উল্লেখ করুন)______________________	1	2

10 আপনাদের খাবার পানির মূল উৎস কি?

নং	উৎসের ধরন	হ্যাঁ	না
1	টিউবওয়েল	1	2
2	পুকুর/নদী/ডোবা / নালা	1	2

		3	বৃষ্টির পানি	1	2
		4	পাইপ/ ট্যাঁপ/ সাপ্লাই পানি	1	2
		5	বিশুদ্ধকৃত/ফিল্টার পানি	1	2
		6	অন্যান্য (উল্লেখ করুন) ________________________	1	2
11	থানায় কি বিদ্যুৎ সংযোগ আছে?	1. হ্যাঁ	2. না		

Part 3B: খানার অন্যান্য আয়ের উৎস

12	থানার কি স্থায়ী কোন আয়ের উৎস আছে?	1. হ্যাঁ	2. না		
14	থানায় কি কোন শিশু উপার্জনকারী আছে?	1. হ্যাঁ	2. না	99. প্রযোজ্য নয়	
15	গত 12 মাসে থানার অন্যান্য আয়ের উৎসগুলি কি ছিল এবং আয়ের পরিমান কত? (একাধিক উত্তর হতে পারে) (Section-2 এ যেসব আয় ধরা হয়েছে তা বাদ দিয়ে অন্তর্ভুক্ত করুন)	আয়ের উৎস			আয়ের পরিমান (টাকায়)
		1. কাজের বিনিময়ে খাদ্য			
		2. সম্পদ থেকে আয়			
		3. ভিজিডি/ দুস্থঃ ভাতা			
		4. বয়স্ক ভাতা			
		5. অবসর ভাতা			
		6. মুক্তিযোদ্ধা ভাতা			
		7. রেমিটেন্স			
		8. উপবৃত্তি/শিক্ষাবৃত্তি			
		9. অন্যান্য ভাতা			
		10. অন্যান্য উৎস (উল্লেখ করুন) _______________			

Part 3C: থানার ঋণ সংক্রান্ত তথ্য

16	থানার কোন সদস্য কি গত একবছরে কোনো ঋণের সাথে যুক্ত ছিলেন?		1. হ্যাঁ	2. না		
	হ্যাঁ হলে নিচের টেবিল পুরন করুন					
	সদস্য কোড	কোন আর্থিক ঋণের সাথে যুক্ত? 1. সমিতি 2. ক্ষুদ্র ঋণদানকারী সংস্থা 3. ব্যাংক থেকে 4. অন্যান্য (উল্লেখ করুন)	সর্বশেষ কতটাকা ঋণ হিসেবে গ্রহণ করেছেন?	বর্তমানে অপরিশোধিত ঋণের পরিমান কত?	গত এক বছরে ঋণের টাকা কোথায় বিনিয়োগ করেছেন? (কোডঃ2)	

কোডঃ2	
1. হাঁস মুরগী পালন	10. মাছ ধরার উপকরন ক্রয়ে
2. গরু ছাগল পালন	11. নৌকা ক্রয়
3. হস্ত শিল্প	12. ভ্যান/রিক্সা ক্রয়
4. কৃষি কাজ	13. ক্ষুদ্র ব্যবসা/দোকান চালানো
5. গৃহ নির্মাণ/মেরামত	14. ব্যবসা
6. বিবাহে	15. কৃষি যন্ত্র ক্রয়
7. ভোগ সামগ্রী ক্রয়ের জন্য	16. ধার/দেনা পরিশোধ
8. শিক্ষা খরচ বাবদ	17. বিদেশে পাঠানো
9. চিকিৎসা বাবদ	18. অন্যান্য

Part 3D: থানার খাদ্য নিরাপত্তা

18	গত এক বছরে আপনি/আপনার পরিবার গড়ে কতদিন তিনবেলা পেটভরে খেতে পেরেছে? (উৎসবের দিন বাদ দিয়ে)	1. প্রায় সবদিন (প্রতিদিন তিনবেলা)<br>2. মাঝেমাঝে (প্রতিদিন তিনবেলা)<br>3. খুব কম (এ বছর ১-৬ দিন, প্রতিদিন তিনবেলা)<br>4. কখনই খায়নি		
19	গত এক একবছরে খাবার স্বল্পতার কারণে গড়ে এমন কতদিন হয়েছে যে আপনি না খেয়ে কাটিয়েছেন?	1. কখনই না<br>2. খুব কম দিনই (বছরে ১-৬ দিন)<br>3. মাঝে মাঝে (বছরে ৭-১২ দিন)<br>4. প্রায় (মাসে কয়েকবার)		
20	গত এক একবছরে খাবার স্বল্পতার কারণে আপনি কি কোন বেলা কম খেয়ে কাটিয়েছেন?	1. কখনই না<br>2. খুব কম দিনই (বছরে ১-৬ দিন)<br>3. মাঝে মাঝে (বছরে ৭-১২ দিন)<br>4. প্রায় (মাসে কয়েকবার)		
21	গত এক একবছরে গড়ে কতবার আপনি বা আপনার পরিবারের কেউ কোন ভাতের স্বল্পতার কারণে রুটি অথবা অন্যকিছু খেয়ে কাটিয়েছেন যদিও? (অসুস্থতার কারনে হলে বাদ দিতে হবে)	1. কখনই না<br>2. খুব কম দিনই (বছরে ১-৬ দিন)<br>3. মাঝে মাঝে (বছরে ৭-১২ দিন)<br>4. প্রায় (মাসে কয়েকবার)		
22	গত এক একবছরে গড়ে কতবার আপনার পরিবার খাবার ছিলনা বলে আত্মীয়স্বজন/প্রতিবেশির কাছে খাবার চেয়েছিল?	1. কখনই না<br>2. খুব কম দিনই (বছরে ১-৬ দিন)<br>3. মাঝে মাঝে (বছরে ৭-১২ দিন)<br>4. প্রায় (মাসে কয়েকবার)		
23	নিজের জমি থেকে যে আয় হয় তা দিয়ে আপনাদের কয়মাস চলে? (এক বছরে)	______ মাস		

Section 4: থানার ব্যয় সংক্রান্ত তথ্য

কঃ 1	কঃ 2	কঃ 3
ক্রঃ নং	খাদ্যের ধরন	ভোগের পরিমান (ভোগ না করে থাকলে “0” লিখুন)
**Part A: খাদ্য (সাপ্তাহিক ব্যয়)**		
**4.1 গত এক সপ্তাহে খাদ্য শস্য**		
1	চাল-মোটা (কেজি)	
	চাল-চিকন (কেজি)	
2	আটা (কেজি)	
3	ময়দা (কেজি)	
4	চিড়া/মুড়ি (কেজি)	

**4.2 গত এক সপ্তাহে ডাল জাতীয় শস্য**		
1	মশুর ডাল (কেজি)	
2	ছোলার ডাল (কেজি)	
3	মাস কালাই (কেজি)	
4	খেসারী (কেজি)	
5	মুগ (কেজি)	
6	বুট (কেজি)	
7	এ্যাংকর (কেজি)	
**4.3 গত এক সপ্তাহে ভোজ্য তেল**		
1	সয়াবিন তেল (লিটার)	
2	সরিষার তেল (লিটার)	
3	ডালডা (কেজি)	
4	ঘি (কেজি)	
5	পাম অয়েল (লিটার)	
6	অন্যান্য তেল (লিটার)_______________	
**4.4 এক সপ্তাহে শাক / সবজি**		
1	লাল শাক (কেজি/আটি)	
2	পুই শাক (কেজি/আটি)	
3	পালং শাক (কেজি/আটি)	
4	কলমি শাক (কেজি/আটি)	
5	অন্যান্য শাক ______________(কেজি/আটি)	
6	অন্যান্য শাক _______________(কেজি/আটি)	
7	অন্যান্য শাক _______________(কেজি/আটি)	
8	আলু (কেজি)	
9	পটল (কেজি)	
10	করলা (কেজি)	
11	ফুলকপি (কেজি/পিছ)	
12	বাধাকপি (কেজি/পিছ)	
13	বেগুন (কেজি)	
14	কচু (কেজি)	
15	লাউ (সংখ্যা)	
16	টমেটো (কেজি)	
17	কাঁচা পেপে (কেজি)	
18	কাঁচা কলা (সংখ্যা)	
19	কাঁচা মরিচ (কেজি)	
20	পেয়াজ (কেজি)	
21	রসুন (কেজি)	
22	আদা (কেজি)	
23	অন্যান্য সবজি___________________ (কেজি)	
24	অন্যান্য সবজি___________________ (কেজি)	
25	অন্যান্য সবজি___________________ (কেজি)	
**Part B: খাদ্য (মাসিক ব্যয়)**		
**4.5 গত এক মাসে মসলা**		
ক্রঃ নং	খাদ্যের ধরন	ভোগের পরিমান (ভোগ না করে থাকলে "0" লিখুন)
কঃ 1	কঃ 2	কঃ 3
1	শুকনা মরিচ (গ্রাম)	
2	হলুদ (গ্রাম)	
3	জিরা (গ্রাম)	
4	দারুচিনি (গ্রাম)	
5	এলাচি (গ্রাম)	
6	অন্যান্য মসলা___________________(গ্রাম)	
7	অন্যান্য মসলা___________________(গ্রাম)	
8	অন্যান্য মসলা___________________(গ্রাম)	
**4.6 গত এক মাসে মাছ, মাংস, ডিম ও দুধ**		
1	বড় মাছ (কেজি)	
2	ছোট মাছ (কেজি)	
3	শুটকি মাছ (কেজি)	
4	গরুর মাংস (কেজি)	
5	মহিষের মাংস (কেজি)	
6	দেশী মুরগীর মাংস (কেজি)	
7	ফার্মের মুরগীর মাংস (কেজি)	
8	হাঁসের মাংস (কেজি)	
9	খাসীর মাংস (কেজি)	
10	ডিম (সংখ্যা)	
11	দুধ (লিটার)	
12	অন্যান্য আমিষ জাতীয় খাবার____________(কেজি)	

13	অন্যান্য আমিষ জাতীয় খাবার___________(কেজি)	
14	অন্যান্য আমিষ জাতীয় খাবার___________(কেজি)	
**4.7 গত এক মাসে ফল -ফলাদি**		
1	কলা (সংখ্যা)	
2	বড়ই(কেজি)	
3	জলপাই(কেজি)	
4	পাকা পেপে(কেজি)	
5	আম (কেজি)	
6	কাঁঠাল (সংখ্যা)	
7	জাম (কেজি)	
8	আপেল (কেজি)	
9	কমলা (সংখ্যা)	
10	আংগুর (কেজি)	
11	পেয়ারা (কেজি)	
12	আনারস (সংখ্যা)	
13	অন্যান্য ফল _________________ (কেজি)	
14	অন্যান্য ফল _________________ (কেজি)	
15	অন্যান্য ফল _________________ (কেজি)	
**4.8 গত এক মাসে অন্যান্য খাবার**		
1	চিনি (কেজি)	
2	গুর (কেজি)	
3	গুঁড়ো দুধ (কেজি)	
4	লবন (কেজি)	
5	নুডুলস (প্যাকেট)	
6	সুজি/সেমাই(কেজি)	
7	কোমল পানীয় (লিটার)	
8	মিষ্টি জাতীয় খাদ্য (কেজি)	
9	শিশুখাদ্য___________________ (কেজি)	
10	চা-পাতা (কেজি)	
11	বিস্কুট (প্যাকেট)	
12	চানাচুর (প্যাকেট)	
13	অন্যান্য _________________ (সংখ্যা)	
14	অন্যান্য _________________ (কেজি)	
15	অন্যান্য _________________ (কেজি)	
**Part C: তামাক জাতীয় দ্রব্য (সপ্তাহিক ব্যয়)**		
**4.9 গত এক সপ্তাহে তামাক জাতীয় দ্রব্য ভোগের পরিমান**		
1	পান (সংখ্যা)	
2	সুপারী (সংখ্যা)	
3	জর্দা (কৌটা)	
4	তামাক (সংখ্যা)	
5	গুল (কৌটা)	
6	বিড়ি (প্যাকেট)	
7	সিগারেট (প্যাকেট)	
8	অন্যান্য _________________ (সংখ্যা)	
9	অন্যান্য _________________ (সংখ্যা)	
10	অন্যান্য _________________ (সংখ্যা)	

Part D: খাদ্য বাদে (মাসিক ব্যয়)		
ক 1	ক 2	ক 3
ক্র:নং	খাত	খরচের পরিমান (মাসিক)
**4.10 গত এক মাসে খাদ্য বাদে ব্যয়**		
1	বাসা ভাড়া	
2	বিদ্যুৎ বিল/ সৌর বিদ্যুৎ	
3	গ্যাস বিল/ গ্যাস সিলিন্ডার	
4	জ্বালানী কাঠ	
5	জ্বালানী তৈল (কেরোসিন, পেট্রোল..)	
6	পরিবহন	
7	স্বাস্থ্য	
8	শিক্ষা(স্কুলের বেতন, কোচিং, প্রাইভেট টিচার ইত্যাদি)	
9	খাতা/কলম/বই/ অন্যান্য শিক্ষাউপকরন	
10	যোগাযোগ ব্যয় (মোবাইল, টেলিফোন ইত্যাদি)	
11	ডিশ বিল/ইন্টারনেট ব্যয়	
12	কসমেটিক ব্যয় (ক্রিম, পাউডার, মেকাপ ইত্যাদি)	
13	চুলে ব্যবহারের তেল (নাড়িকেল তেল)	
14	পরিষ্কার ও পরিচ্ছন্নতা (সাবান, টুথপেস্ট, ডিটারজেন্ট, শ্যাম্পু ইত্যাদি)	
15	লন্ড্রি	
16	সমিতির কিস্তি/ক্ষুদ্রঋনের কিস্তি	
17	সেলুন/পার্লার	
18	অন্যান্য(নির্দিষ্টকরেবলুন)	

Part E: খাদ্যবাদে (বার্ষিক ব্যয়)		
**4.11 গত এক বছরে খাদ্য বাদে ব্যয়**		
ক1	ক2	ক3
ক্র:নং	খাত	খরচের পরিমান (বার্ষিক)
1	পোশাক	
2	জুতা	
3	লেপ/তোষক/বালিশ/চাদর/বিছানা	
4	বিনোদন / ধর্মীয় অনুষ্ঠান	
5	গৃহ নির্মাণ/গৃহ সংস্কার	
6	আসবাবপত্র	
7	ইন্সুরেন্স প্রিমিয়াম	
8	গৃহপরিচালক / গৃহপরিচারিকার বেতন	
9	উপহার প্রদান	
10	দান/ফিতরা	
11	ভ্রমন	
12	অন্যান্য(নির্দিষ্টকরেবলুন)	

Section 5: খানার সদস্যদের বিগত তিন মাসে অসুস্থতা, চিকিৎসা এবং তার ব্যয় সম্পর্কিত তথ্য (একাধিক অসুস্থতার তথ্য সদস্য কোড সহ ভিন্ন ভিন্ন সারিতে লিখুন)

গত তিন মাসে আপনি বা আপনার খানার কোন সদস্য কি কোন রোগের লক্ষণ/ কোন রোগে আক্রান্ত হয়েছিল কি? ......1. হ্যাঁ...... 2. না (হ্যাঁ হলে নিম্নোক্ত টেবিল টি পূরণ করুন)

ক1	ক2	ক3	ক4	ক5	ক6		ক7		ক8	ক9										ক10	ক11	ক12	ক13	ক14					
(সদস্য কোড)	ঐ সদস্যের রোগের কোড লিখুন*	আপনি/ঐ সদস্য ঐ অসুখে কতদিন ভুগেছিলেন?	আপনি/ঐ সদস্য কি ঐ অসুখের জন্য স্বাস্থ্যসেবা নিয়েছিলেন? কোডঃ 1. হ্যাঁ, 2. না (উত্তর যদি না হয় তবে শুধু ক5 অংশ প্রযোজ্য)	যদি চিকিৎসা না নিয়ে থাকেন, কি কারনে নেননি?<br>1. জটিল কোনো সমস্যাছিলনা<br>2. চিকিৎসার খরচ অত্যধিক<br>3. পর্যাপ্ত অর্থ ছিলনা<br>4. স্বাস্থ্যকেন্দ্রের দূরত্ব খুব বেশি<br>5. স্বাস্থ্যকেন্দ্রে নিয়ে যাওয়ার কেউ ছিল না<br>6. স্বাস্থ্যকেন্দ্রের অবস্থান জানেননা<br>7. অন্যান্য (উল্লেখ করুন....)	আপনি কার কাছ থেকে চিকিৎসা নিয়েছিলেন?<br>1. মেডিকেল কলেজ হাসপাতাল<br>2. বিশেষায়িত হাসপাতাল<br>3. রেফার্ড জেলাসদর হাসপাতাল (এসএসকে)<br>4. অন্যান্য জেলাসদর হাসপাতাল<br>5. উপজেলা হেলথ কমপ্লেক্স<br>6. ইউনিয়ন সাবসেন্টার/ কমিউনিটি ক্লিনিক/রুরাল ডিসপেন্সারী<br>7. মাতৃ ও শিশু সেবাকেন্দ্র<br>8. বেসরকারী ক্লিনিক/হাসপাতাল<br>9. এনজিও ক্লিনিক/হাসপাতাল<br>10. এনজিও স্বাস্থকর্মী<br>11. সরকারি স্বাস্থ্য কর্মী<br>12. বেসরকারী চিকিৎসক (স্বীকৃত ডিগ্রী)<br>13. বেসরকারী চিকিৎসক (অনানুষ্ঠানিক ডিগ্রী)<br>14. কবিরাজ/হোমিওপ্যাথিক<br>15. নিজ চিকিৎসা<br>16.ঔষধের দোকান<br>17.অন্যান্য (উল্লেখ করুন)		আপনার কি ঐ সমস্যা বা অসুখের জন্য হাসপাতালে ভর্তি হতে হয়েছিলো? কোডঃ 1. হ্যাঁ 2. না (না হলে ক.9 এ যান)	যদি হ্যাঁ হয় তবে, কতদিন হাসপাতালে ভর্তি ছিলেন? (পূর্নদিবস)	চিকিৎসা কি এসএসকে আওতাধীন 1. হ্যাঁ 2. না 99. প্রযোজ্য নয়	চিকিৎসা বাবদ খরচ (যে ব্যয়টি প্রযোজ্য হবে না, সেক্ষেত্রে ‘0’ লিখুন)										অসুস্থতা জনিত কারনে কতদিন কাজে অনুপস্থিত ছিলেন (পূর্নদিবস)	বিগত 3 মাসের মধ্যে অসুস্থতাসহ কতদিন কাজে উপস্থিত ছিলেন? (পূর্ন দিবস) (প্রযোজ্য না হলে “99” লিখুন)	গত 3 মাসের মধ্যে অসুস্থতার কারণে স্থায়ীভাবে/ সাময়িক ভাবে কোনো কাজ ছেড়েছেন কি? 1. হ্যাঁ, 2.না 99. প্রযোজ্য নয়	গত 12 মাসের মধ্যে অসুস্থতার কারণে স্থায়ীভাবে স্কুল ছাড়তে হয়েছিল কি? 1. হ্যাঁ, 2. না 99. প্রযোজ্য নয়	আপনি চিকিৎসার খরচ কিভাবে জোগাড় করেছিলেন?<br>1. নিয়মিত আয়<br>2. পরিবারের সঞ্চয়<br>3. এস.এস.কে<br>4. বীমা<br>5. নিজস্ব জিনিসপত্র বিক্রি<br>6. গাছ/কৃষিপণ্য/গৃহপালিতপ্রাণীবিক্রি<br>7. স্থায়ী সম্পত্তি বিক্রি<br>8. জমি বা সম্পত্তি বন্ধক<br>9. মহাজনের কাছ থেকে টাকা ধার<br>10. বন্ধু বা আত্মীয় স্বজনদের কাছ থেকে সাহায্য<br>11. আত্মীয়/বন্ধু/সহকর্মীদের কাছ থেকে ধার<br>12. কোন প্রতিষ্ঠান/সমিতি থেকে ঋণ গ্রহণ<br>13.অন্যান্য (নির্দিষ্টকরুন)					
					ক্রমিক নং	চিকিৎসার উৎস				পরামর্শ ফি	ঔষধ	বিছানা	পরীক্ষা খরচ	যাতায়াত	বকশিশ	এটেন্ডেন্ট	প্যাকেজ	অপারেশন	অন্যান্য					উৎস-1	টাকা	উৎস-2	টাকা	উৎস-3	টাকা
					1.																								
					2.																								
					3.																								
					1.																								
					2.																								
					3.																								
					1.																								
					2.																								
					3.																								
					1.																								
					2.																								
					3.																								

*1=সর্দি কাশি, 2=জ্বর,3=টাইফয়েড, 4=ডায়রিয়া/পাতলা পায়খানা/আমাশয়, 5=বমি, 6=হাঁপানি/শ্বাসকষ্ট, 7=ছোট কাটাছেঁড়া,8=হাড়ভাঙ্গা 9=জন্ডিস, 10=দুর্বলতা, 11=কৃমি, 12=ব্যাথা, 13=চর্মরোগ, 14=খিঁচুনি, 15=বুকজ্বালা, 16=উচ্চ রক্তচাপ, 17=ডায়াবেটিস, 18=যক্ষ্মা, 19=কুষ্ঠরোগ, 20=পক্ষাঘাত/প্যারালাইসিস, 21=বাত, 2=বাতজ্বর 23= ক্যান্সার, 24= সন্তান প্রসব, 25= গর্ভসংক্রান্ত সমস্যা 26= মানসিক সমস্যা, 27= অন্যান্য (নির্দিষ্ট করুন)

 Version date: 29.05.18

Section 6: খাদ্য নিরাপত্তার উপর অসুস্থতার প্রভাব<br>(যদি খানার কোনো সদস্য অসুস্থতায় না ভোগেন তবে পরবর্তী সেকশন থেকে প্রশ্ন করুন)				
ক্রঃনং	প্রশ্ন	উত্তরের ধরন		
1	গত 3 মাসের মধ্যে খানার কোনো সদস্যের অসুস্থতার খরচের জন্য আপনার পরিবারের খাদ্য গ্রহণের খরচ কমাতে হয়েছিল কি?	1. হ্যাঁ	2. না	৭৭. প্রযোজ্য নয়
2	গত 3 মাসের মধ্যে খানার কোনো সদস্যের অসুস্থতার খরচের জন্য আপনার পরিবারের খাদ্য বাদে অন্যান্য খরচ (যেমনঃ শিক্ষা, পোষাক, আসবাব ইত্যাদি) কমাতে হয়েছিল কি?	1. হ্যাঁ	2. না	৭৭. প্রযোজ্য নয়

Section 7: স্বাস্থ্যসেবার ব্যয়ের প্রভাব ও এস.এস.কে সম্পর্কিত জিজ্ঞাসা

ক্রঃনং (ক1)	প্রশ্ন (ক2)	উত্তরের ধরন (ক3)	
1	আপনার কি মনে হয় যে বাংলাদেশের অনেকেই চিকিৎসার খরচ যোগাতে গিয়ে অর্থনৈতিক ভাবে ক্ষতির সম্মুখীন হন?	1. হ্যাঁ	2. না
2	আপনার কি মনে হয় যে আপনার আশে পাশের অনেকেই প্রতি মাসের কিছুদিন অসুস্থতা নিয়ে কাজ করেন?	1. হ্যাঁ	2. না
3	কেউ অসুস্থ হলে অনেক প্রতিষ্ঠান তার সমগ্র চিকিৎসার ব্যয়বহন করে সে সম্পর্কে আপনি কি জানেন?	1. হ্যাঁ	2. না
4	আপনি কি জীবন বীমা/লাইফ ইনস্যুরেন্স সম্পর্কে শুনেছেন?	1. হ্যাঁ	2. না
5	আপনি কি স্বাস্থ্য বীমা/হেলথ ইনস্যুরেন্স সম্পর্কে শুনেছেন?	1. হ্যাঁ	2. না
6	আপনি কি জানেন এই এলাকায় গরীব লোকদের স্বাস্থ্য সুরক্ষার জন্য কর্মসুচী/এসএসকে চালু আছে?	1. হ্যাঁ	2. না
7	এস.এস.কে সেবা কেন্দ্র থেকে কিভাবে সেবা দেওয়া হয় তা কি আপনি কি জানেন?	1. কার্ডের মাধ্যমে<br>2. টাকার বিনিময়ে<br>3. জানিনা	
8	আপনি কি জানেন এস.এস.কে সেবা কেন্দ্র থেকে কতটি রোগের সেবা দেওয়া হয়?	1. হ্যাঁ<br>(হ্যাঁ হলে জানতে চান কতটি) _____ টি	2. না
9	এস.এস.কে থেকে চিকিৎসার জন্য কি ধরনের খরচ বহন করা হয়?<br>(একাধিক উত্তর হতে পারে)	1. বিনামুল্যে ঔষধ<br>2. বিনামুল্যে পরীক্ষা/ডায়াগনস্টিক<br>3. রেফারেল সুবিধা<br>4. জেলা হাসপাতালে পাঠালে তার খরচ	
10	এস.এস.কে সম্পর্কে আপনি কোন মাধ্যম থেকে জেনেছেন?<br>(একাধিক উত্তর হতে পারে)	1. এলাকার অন্যের কাছে<br>2. এস.এস.কে এর প্রতিনিধির কাছে<br>3. পরিচিত জনের কাছে<br>4. এলাকার জনপ্রতিনিধির কাছে<br>5. মাইকিং এর মাধ্যমে<br>6. পোস্টার থেকে<br>7. বিলবোর্ড থেকে<br>8. টিভি স্ক্রল বিজ্ঞাপন থেকে<br>9. ভিডিও দেখে<br>10. অন্যান্য (উল্লেখ করুন)	

Part 7A: এস.এস.কে সেবা গ্রহণ সংক্রান্ত তথ্য (এসএসকে মেম্বারদের জন্য)

11	এস.এস.কে কার্ড নাম্বারঃ (কার্ড দেখে পুরুন করুন)	|___|___||___|___||___|___||___|___||___|___||___|___||___|___||___|___|___|	
12	কার্ড গ্রহণের তারিখ	|___|___|:|___|___|:|___|___|___|___| (দিন / মাস / বছর)	
13	কার্ড যে ব্যক্তির নামে তার সদস্য কোড লিখুন (Section 2 থেকে)	|___|___| সদস্য কোড	
14	কখনও কি এস.এস.কে কার্ড ব্যবহার করেছিলেন? না করলে কেন করেননি?	1. হ্যাঁ	2. না (পরবর্তী সেকশনে যান)
15	কতবার ব্যবহার করেছিলেন?	__________ বার	

Section 8: খানা সদস্যদের বর্তমান স্বাস্থ্যের অবস্থা সংক্রান্ত প্রশ্ন

(নিচের প্রতিটি গ্রুপে তিনটি করে বক্স আছে, যেকোনো একটি বক্সে টিক চিহ্ন দিন। সেই বক্সটিতেই টিক চিহ্ন দিবেন যেটি আপনার শারীরিক অবস্থা সঠিকভাবে নির্দেশ করে।)

সদস্য কোডঃ											
A	**হাঁটা চলা**<br>1. হাঁটা চলায় আমার কোনো অসুবিধা হয়না<br>2. হাঁটা চলায় আমার কিছুটা অসুবিধা হয়<br>3. বিছানা থেকে উঠতে পারিনা										
B	**নিজের যত্ন নিজে নেয়া**<br>1. নিজের যত্ন নিতে আমার কোন অসুবিধা হয়না<br>2. নিজে গোসল করতে বা কাপড় পরতে আমার কিছুটা অসুবিধা হয়<br>3. নিজে গোসল করতে বা কাপড় পরতে পারিনা										
C	**সাধারন কাজকর্মঃ যেমন টুকিটাকি কাজ, পড়াশুনা, বাড়িঘরের কাজ, অবসরের কাজ ইত্যাদি**<br>1. সাধারন কাজকর্ম করতে আমার কোন অসুবিধা হয়না<br>2. সাধারন কাজকর্ম করতে আমার কিছুটা অসুবিধা হয়<br>3. সাধারন কাজকর্ম করতে পারিনা										
D	**গায়ে ব্যথা/ গায়ে খারাপ লাগা**<br>1. সাধারন কাজকর্ম করতে আমার কোন অসুবিধা হয়না<br>2. সাধারন কাজকর্ম করতে আমার কিছুটা অসুবিধা হয়<br>3. সাধারন কাজকর্ম করতে পারিনা										
E	**দুশ্চিন্তা/মনমরা ভাব**<br>1. আমার দুশ্চিন্তা বা মনমরা ভাব নেই<br>2. আমার কিছুটা দুশ্চিন্তা বা মনমরা ভাব আছে<br>3. আমার খুব দুশ্চিন্তা হচ্ছে বা আমি খুব মনমরা আছি										
F<br>আজ আপনার শরীরের অবস্থা স্কেলে চিহ্নিত করুন<br>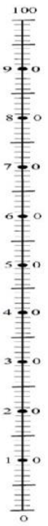	শরীরের ভাল না খারাপ অবস্থা আছে তা বুঝানোর জন্য নিম্নে একটি স্কেল আছে। শরীর পুরোপুরি ভালো হলে এই স্কেলে 100 তে দাগ দিয়ে বুঝাতে হবে এবং একেবারেই খারাপ হলে 0 তে দাগ দিয়ে বুঝাতে হবে।<br><br>আপনার মতে আজ আপনার শরীর কতটা ভাল বা খারাপ তা নিম্নের স্কেল দিয়ে বুঝান । স্কেলের যে পয়েন্টটা আপনার শরীরের আজকের অবস্থার সঙ্গে মিলে, সেই পয়েন্টটিতে ক্রস চিহ্ন দিন।										

আপনাকে অনেক ধন্যবাদ

 Version date: 29.05.18
